# Magnetic dipole effects on unsteady flow of Casson-Williamson nanofluid propelled by stretching slippery curved melting sheet with buoyancy force

**DOI:** 10.1038/s41598-023-39354-5

**Published:** 2023-08-07

**Authors:** Pradeep Kumar, Basavarajappa Nagaraja, Felicita Almeida, Abbani Ramakrishnappa AjayKumar, Qasem Al-Mdallal, Fahd Jarad

**Affiliations:** 1 Department of Mathematics, School of Engineering, Presidency University, Rajanakunte, Yelahanka, Bengaluru, Karnataka 560064 India; 2 Department of Mathematics, KNS Institute of Technology, Thirumenahalli, Yelahanka, Bangalore, 560064 India; 3 Department of Mathematical Sciences, UAE University, P.O. Box 17551, Al-Ain, United Arab Emirates; 4 Department of Mathematics, Çankaya University, 06790 Ankara, Turkey; 5 Department of Medical Research, China Medical University, Taichung, 40402 Taiwan

**Keywords:** Applied mathematics, Fluid dynamics

## Abstract

In particular, the Cattaneo-Christov heat flux model and buoyancy effect have been taken into account in the numerical simulation of time-based unsteady flow of Casson-Williamson nanofluid carried over a magnetic dipole enabled curved stretching sheet with thermal radiation, Joule heating, an exponential heat source, homo-heterogenic reactions, slip, and melting heat peripheral conditions. The specified flow's partial differential equations are converted to straightforward ordinary differential equations using similarity transformations. The Runge–Kutta–Fehlberg 4-5th order tool has been used to generate solution graphs for the problem under consideration. Other parameters are simultaneously set to their default settings while displaying the solution graphs for all flow defining profiles with the specific parameters. Each produced graph has been the subject of an extensive debate. Here, the analysis shows that the thermal buoyancy component boosts the velocity regime. The investigation also revealed that the melting parameter and radiation parameter had counterintuitive effects on the thermal profile. The velocity distribution of nanofluid flow is also slowed down by the ferrohydrodynamic interaction parameter. The surface drag has decreased as the unsteadiness parameter has increased, while the rate of heat transfer has increased. To further demonstrate the flow and heat distribution, graphical representations of streamlines and isotherms have been offered.

## Introduction

Modern study in the topic is being encouraged by the expanded applications of multiphase flow of various fluid types across continuously extended surfaces. One such example is the movement of fluid across a curved stretched sheet. With a change in flow steering settings, the stretched sheet's curved form alters how the flow behaves. This subject of research has benefited from the contributions of several experts from throughout the world. In their exploratory study of the mixed convective flow of water-based nanofluids across an extended curved surface, Hayat et al.^1^ found convergent series solutions. The impact of an exponential, space-dependent heat source on the Casson fluid flow across a stretched, curved sheet has been researched by Nagaraja and Gireesha^2^. The stretching pace and flow model is time-independent in the aforementioned experiments. However, the sheet elongation may begin briefly or unsteadily in many engineering and technology challenges. Therefore, the need for testing the unsteady flow across stretched geometry has increased. For the dual stratified flow of Casson fluid Chen et al^3^ analysed heat and mass flux using Fourier's and Fick's laws. Waqas et al^4–6^ looked into dynamics of various non-Newtonian fluid under different circumstances. Ramzan et al^7^ looked into the mixed convective flow Casson fluid in presence of gyrotactic microbes between two concentric cylinders. Nasir et al^8^ analysed non-linear convective -radiative flow of Oldroyd B non-Newtonian fluid when subjected Robin’s boundary condition. They have concluded that increasing relaxation parameter diminishes velocity profile. Further Nasir et al^9^ have also interpreted the results for Casson nanofluid over a vertical convective surface. The unsteady flow across curved geometry has been further studied by several other researchers^10,11^.

The extensive research on non-Newtonian fluids meets a lot of the demands of contemporary industry. Specifically, the non-Newtonian nanofluid flow over continually stretched geometry. It has several uses in the polymer industry, thinning, and many other production sectors. As a result, non-Newtonian nanofluids are the principal subject of current study. Numerous scholars have investigated non-Newtonian nanofluid models such as Casson, Carreau, Maxwell, Jeffrey, etc. The novel topic of study that has already attracted the attention of many scientists is the merging of non-Newtonian fluid models like Casson-Micropolar, Carreau-Yasuda, and Casson-Carreau. In their study of the time-dependent 3-D flow of Casson-Carreau fluid across a continuously extended surface, Raju and Sandeep^12^ discovered that the heat and mass transfer rates in Casson fluid are higher than those in Carreau fluid. Amjad et al.^13^ examined the Casson micropolar nanofluid flow over a porous curved stretching sheet and came to the conclusion that the curvature parameter reduces the microrotation profile. A comparison study of the 3-D Casson-Carreau fluid flow across a porous curved stretched surface was done by Akolade and Tijani^14^. An investigation of the MHD flow of Casson-Williamson fluid across a magnetically enhanced stretching surface with numerous slip boundaries has been provided by Humane et al.^15^. They have illustrated how the thermal radiation and magnetic field influence the thermal profile.

The conversion of thermal radiation from fluid motion into electromagnetic radiation has applications in a wide range of industries, including solar energy products, car radiators, thermal power plants, and many more. A study by Naveed et al.^16^ examined the impact of thermal radiation on the flow of micropolar fluid powered by a curved stretching sheet and came to the conclusion that by enhancing the radiation parameter, the performance of the thermal panel is enhanced. Megahed et al.^17^ investigated the problem of boundary layer MHD flow produced by an unsteady stretching sheet using variable fluid properties, heat flux, and thermal radiation. They concluded that as the radiation parameter increases, the thermal profile decreases near the boundary and then improves. Williamson fluid flow across stretching and shrinking geometry with thermal radiation has been examined by Ibrahim and Negera^18^ in their work. Waqas et al^19^ studied the radiative effects on bioconvective micropolar nanofluid flow over a stretched surface whereas Pasha et al^20^ recorded the impact of radiation effects tangent hyperbolic flow under the consideration of Soret Dufour effects.

Dipole is an abstract system that makes field estimations using a challenging charge mechanism. Typically, a magnetic dipole serves as the source of a static magnetic field. It is undeniable that the magnetic dipole phenomenon is connected to the magnetic field and is thus frequently used in medicine. A magnetic dipole has advantages in NMR spectroscopy and magnotherapy. Yasmeen et al.^21^ analysed the magnetic dipole for homo-heterogenic processes when ferrous particles are suspended in the carrier fluid. Hayat et al.^22^ conducted more research on this dipole contribution to Williamson fluid. Gowda et al.^23^ investigated how a magnetised ferro fluid magnetic dipole might affect an extended sheet. By accelerating the transfer of heat, Stefan blowing presence illustrates its significance. Zeeshan and Majeed^24^ investigated how a magnetic dipole affected the flow and heat transfer of Jeffery fluid across a stretched sheet. They have explained how the temperature and velocity of ferromagnetic interactions change. The study on the magnetic dipole influence on the unsteady flow of various fluids across stretching geometry has been supported by several more researcher^25^.

Streams in the ocean, solar receivers, heterogeneously pushed air flows, and etc., are examples of naturally occurring phenomena where mixed convective (which includes both natural and forced) flows have a variety of uses. One such characteristic of mixed convection is the buoyancy force brought on by temperature and density variations. In their research on the flows of Walter's-B fluid and tangent hyperbolic nanofluid^26,27^, Khan et al.^28^ took non-linear mixed convection into account.

Numerous chemically reactive structures incorporate homo-heterogenic processes, including catalysis, combustion, and biological systems. A really complicated relationship exists between homo-heterogenic forms of responses. According to Imtiaz et al.^29^, homo-heterogenic reactions have an impact on the time-dependent flow over curved geometry. According to their findings, concentration distribution increases for heterogenic reaction parameters and decreases for homogenic reaction parameters. Pal and Mandal^30^ investigated the effect of homo-heterogeneous reactions on the flow of CNT nanofluid across a stretched plate. The study on homo-heterogenic reaction’s impact on various fluids over curved stretching geometry has been supported by several other researchers^31,32^. Numerous renowned researchers have taken into account the slip flow and melting heat phenomena process at the interface for various fluid flows^33,34^.

To examine the mechanism of heat transport under vivid conditions, a variety of models have been proposed, one of which is the Fourier convention model for heat conduction. However, this idea encountered a problem since it produced a parabolic thermal field and defied the causality principle. Thus, the thermal relaxation time came into play, allowing the transmission of heat through the propagation of thermal waves at a limited pace. The uses of this concept ranged from nanoliquid flow to skin blisters brought on by burning. In order to maintain the notion of the material-invariant, Christov^35^ altered the Cattaneo law by adding a new term that combines the derivative of time with the derivative of Oldroyd's upper convection. Furthermore, Ali et al.^36^ used the Cattaneo-Christov paradigm, which revealed the outcomes of the Soret-Dufour effects. According to their inquiry, the velocities are known to improve when the parameter related to rotating fluid is enlarged. Further several authors^37,38^ carried out the study for the nanofluid and hybrid nanofluid flow in presence of Cattaneo–Christov heat flux. Ahmad et al^39^ discussed double diffusion for the flow Eyring–Powell -liquid. Wang et al^40^ deliberated the Cattaneo–Christov heat-mass transfer for a third-grade fluid flow over a stretched surface and homotopy scheme was implemented to obtain the convergent solutions.

An examination of the above recent literature reveals that the present investigation which models the unsteady flow of a Casson-Williamson nanofluid, is innovative and an advancement in the field since combination of flow of two different non-Newtonian fluid models has many applications in the manufacturing sector. The modelling has been done in curvilinear coordinates, under the circumstances of the buoyancy effect, the Cattaneo-Christov heat flux model, thermal radiation, homo-heterogenic processes, Joule heating, exponential heat production, and the magnetic dipole moment. The slip and melting heat conditions are considered at the boundary of the stretching surface. When it comes to curved shape, considerable effects, and optimal peripheral circumstances, the innovative combination of Casson nanofluid and Williamson nanofluid in the current work is relatively worth analysing. The results of the present study are significant and welcome the further research in the field due to the showcase of lucid behavioural changes of all flow profiles for imperative parameters of engineering interest.

## Mathematical articulation

**Figure 1:**
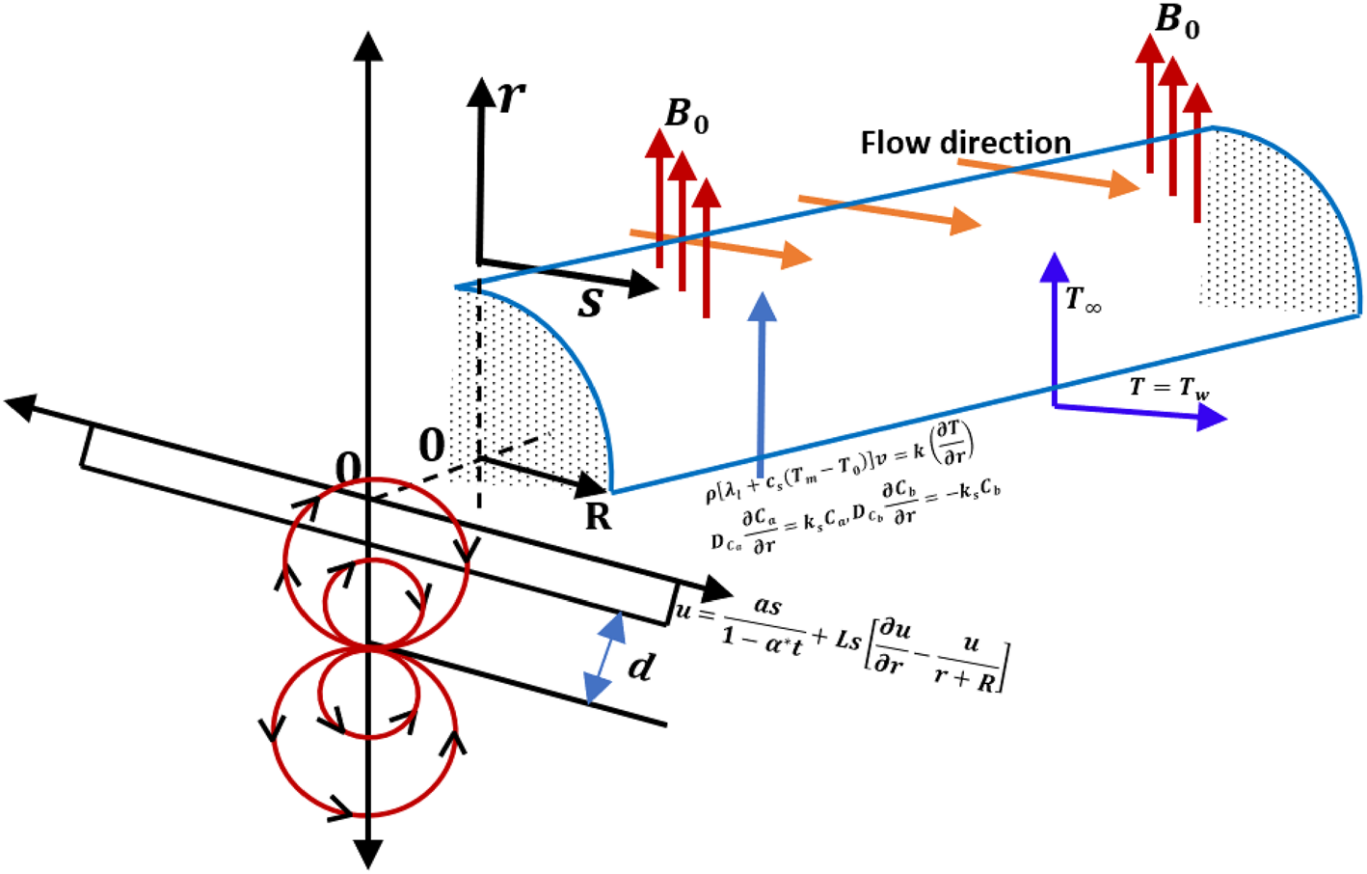
Graphical delineation of flow problem.

Consider an unsteady flow of Casson-Williamson nanofluid along a curved stretching sheet. The sheet is vulnerable to stretching around a semicircle of radius R by two equal and opposite pressures applied along the s-orientation while maintaining the origin stationary and the r-orientation normal to it as portrayed in Fig. 1. Allowing for ${u}_{w}\left(s\right)=\frac{as}{1-{\alpha }^{*}t}$ as the sheet's stretching speed, where $t$ is time, $a$ is the stretching rate, and ${\alpha }^{*}$ is a constant with dimension reciprocal of time. When analyzing the fluid flow behavior, the impact of the magnetic dipole moment is taken into account. In the r direction, the magnetic field ${B}_{m}=\frac{{B}_{0}}{\sqrt{(1-{\alpha }^{*}t)}}$ is applied. To examine the flow properties of the aforementioned nanofluid, the influence of buoyancy is taken into consideration. To study the thermal behavior of the flow, the effects of thermal radiation, Joule heating, and an exponential space dependent heat source are taken into account in the energy equation. In order to fully understand how it affects the flow, the Cattaneo-Christov heat flux model has been used. With the help of the slip condition, the melting surface's boundary is enhanced.

To explain the mass transfer operation, two chemical samples $A$ and $B$ at corresponding concentrations ${C}_{a}$ and ${C}_{b}$ are studied for their homo-heterogenic reactions. $A+2B\to 3B$ with a rate of ${k}_{c}{C}_{a}{C}_{b}^{2}$ is the homogenic reaction on the carrier surface, whereas $A\to B$ with a rate of ${k}_{s}{C}_{a}$ is the heterogenic reaction.

These ideas lead to the formulation of the flow anchoring equations as^29^.

1 $$\frac{\partial }{\partial r}\{(R+r)v\}+R\frac{\partial u}{\partial s}=0,$$

2 $$\frac{\rho }{r+R}{u}^{2}=\frac{\partial p}{\partial r},$$

3 $$\frac{\partial u}{\partial t}+v\frac{\partial u}{\partial r}+\frac{R}{r+R}u\frac{\partial u}{\partial s}+\frac{uv}{r+R}=-\frac{R}{\rho (r+R)}\frac{\partial p}{\partial s}+\nu \left[\left(1+\frac{1}{\beta }\right)\left(\frac{{\partial }^{2}u}{\partial {r}^{2}}+\frac{1}{r+R}\frac{\partial u}{\partial r}-\frac{u}{{\left(r+R\right)}^{2}}\right)+\sqrt{2}\Gamma \left\{\frac{\partial u}{\partial r}\frac{{\partial }^{2}u}{\partial {r}^{2}}+\frac{1}{r+R}\left\{{\left(\frac{\partial u}{\partial r}\right)}^{2}-u\frac{{\partial }^{2}u}{\partial {r}^{2}}\right\}+\frac{{u}^{2}}{{\left(r+R\right)}^{3}}-\frac{2u}{{\left(r+R\right)}^{2}}\frac{\partial u}{\partial r}\right\}\right]+\frac{{\mu }_{0}M}{\rho }\frac{\partial H}{\partial s}+g[{\beta }_{T}\left(T-{T}_{\infty }\right)]-\frac{\sigma }{\rho }{B}_{m}^{2}u,$$

4 $$\frac{\partial T}{\partial t}+v\frac{\partial T}{\partial r}+\frac{Ru}{r+R}\frac{\partial T}{\partial s}=\alpha \left(\frac{{\partial }^{2}T}{\partial {r}^{2}}+\frac{1}{r+R}\frac{\partial T}{\partial r}\right)+\tau \left[{D}_{{C}_{a}}\left(\frac{\partial {C}_{a}}{\partial r}\frac{\partial T}{\partial r}\right)+\frac{{D}_{T}}{{T}_{\infty }} {\left(\frac{\partial T}{\partial r}\right)}^{2}\right]-\frac{1}{\rho {c}_{p}}\frac{1}{r+R}\frac{\partial \left[\left(r+R\right){q}_{r}\right]}{\partial r}+ \frac{{Q}_{0}}{\rho {c}_{p}}\left(T-{T}_{\infty }\right) {\mathrm{e}}^{\left(-\sqrt{\frac{a}{\nu }} r\right)}+\frac{1}{\rho {c}_{p}}\left[u\frac{\partial H}{\partial s}+\frac{\partial H}{\partial r}\right]{\mu }_{0}T\frac{\partial M}{\partial T} -{\lambda }_{1}\left[{v}^{2}\frac{{\partial }^{2}T}{\partial {r}^{2}}+{u}^{2}{\left(\frac{R}{r+R}\right)}^{2}\frac{{\partial }^{2}T}{\partial {s}^{2}}+\left(v\frac{\partial v}{\partial r}+\frac{uR}{r+R}\frac{\partial v}{\partial s}\right)\frac{\partial T}{\partial s}+\left(\frac{vR}{r+R}\frac{\partial u}{\partial r}+u{\left(\frac{R}{r+R}\right)}^{2}\frac{\partial u}{\partial s}\right)\frac{\partial T}{\partial s}+\frac{2uvR}{r+R}\frac{{\partial }^{2}T}{\partial r\partial s}\right]+\frac{\sigma }{\rho {c}_{p}}{B}_{m}^{2}{u}^{2},$$

5 $$\frac{\partial {C}_{a}}{\partial t}+v\frac{\partial {C}_{a}}{\partial r}+\frac{Ru}{r+R}\frac{\partial {C}_{a}}{\partial s}={D}_{{C}_{a}}\left(\frac{{\partial }^{2}{C}_{a}}{\partial {r}^{2}}+\frac{1}{r+R}\frac{\partial {C}_{a}}{\partial r}\right)+\frac{{D}_{T}}{{T}_{\infty }}\left(\frac{{\partial }^{2}T}{\partial {r}^{2}}+\frac{1}{r+R}\frac{\partial T}{\partial r}\right)-{k}_{c}{C}_{a}{C}_{b}^{2},$$

6 $$\frac{\partial {C}_{b}}{\partial t}+v\frac{\partial {C}_{b}}{\partial r}+\frac{Ru}{r+R}\frac{\partial {C}_{b}}{\partial s}={D}_{{C}_{b}}\left(\frac{{\partial }^{2}{C}_{b}}{\partial {r}^{2}}+\frac{1}{r+R}\frac{\partial {C}_{b}}{\partial r}\right)+\frac{{D}_{T}}{{T}_{\infty }}\left(\frac{{\partial }^{2}T}{\partial {r}^{2}}+\frac{1}{r+R}\frac{\partial T}{\partial r}\right)+{k}_{c}{C}_{a}{C}_{b}^{2},$$

associated auxiliary conditions are^33^

7 $$u=\frac{as}{\left(1-{\alpha }^{*}t\right)}+Ls\left[\frac{\partial u}{\partial r}-\frac{u}{r+R}\right], k\left(\frac{\partial T}{\partial r}\right)=\rho \left[{\lambda }_{l}+{c}_{s}\left({T}_{m}-{T}_{0}\right)\right]v, T={T}_{w}, {D}_{{C}_{a}}\frac{\partial {C}_{a}}{\partial r}={k}_{s}{C}_{a}, {D}_{{C}_{b}}\frac{\partial {C}_{b}}{\partial r}=-{k}_{s}{C}_{a}\,\,\mathrm{ at }\,\,r=0, u\to 0, v\to 0, \frac{\partial u}{\partial r}\to 0, T\to {T}_{\infty }, {C}_{a}\to {C}_{0}, {C}_{b}\to 0 \,\,as\,\, r\to \infty .$$

Here $\left(u, v\right)$-velocities along $\left(s, r\right)$-orientations, $p$-pressure, $\rho$-density, $\nu$-kinematic viscosity, $\beta$-Casson parameter, $\Gamma$-material time constant, $g$-acceleration, ${\beta }_{T}$-coefficient of thermal expansion, ${\mu }_{0}$-magnetic permeability, $\mathrm{M}$-magnetization, $\mathrm{H}$-magnetic field, $\sigma$-electrical conductivity, ${B}_{0}$-constant magnetic field, $T$-temperature, $\alpha$-thermal diffusivity, ${c}_{p}$-specific heat, $\tau$-ratio of the effective heat capacity, ${q}_{r}$-radiative heat flux, ${Q}_{0}$-space dependent heat source, ${\lambda }_{1}$-relaxation time of heat, ${D}_{{C}_{a}}$-diffusion coefficient of $A$, ${D}_{{C}_{b}}$-diffusion coefficient of $B$, ${T}_{w}$-temperature of the fluid at the surface, ${T}_{\infty }$-ambient fluid temperature, ${D}_{T}$-thermophoretic diffusion coefficient, $\left({k}_{c}, {k}_{s}\right)$-rate constants, $Ls$-velocity slip coefficient, ${\lambda }_{l}$-latent heat of the fluid, ${c}_{s}$-heat capacity of the solid surface, ${T}_{m}$-melting temperature, ${T}_{0}$-temperature of the solid surface and $k$-thermal conductivity.

Given by the Rosseland estimation, the radiative heat flow is,

8 $${q}_{r}=-\frac{4{\sigma }^{*}}{3{k}^{*}}\frac{\partial {T}^{4}}{\partial r}=-\frac{16{\sigma }^{*}{T}_{\infty }^{3}}{3{k}^{*}}\frac{\partial T}{\partial r},$$

where the Stefan-Boltzmann constant and the coefficient of mean absorption, respectively, are written as ${\sigma }^{*}$ and ${k}^{*}$.

### Magnetic dipole

The apparent magnetic dipole and its scalar strength $\Phi$ cause the magnetic field to have the following effects on the liquid stream:

9 $$\Phi =\frac{\gamma }{2\pi }\left\{\frac{s}{{s}^{2}+{\left(r+d\right)}^{2}}\right\},$$

where $d$ is the distance between the dipoles and $\gamma$ is the intensity of the magnetic field at the source. The characteristics of associated magnetic field $H$^41^ are as follows

10 $${H}_{r}=-\frac{\partial\Phi }{\partial r}=\frac{\gamma }{2\pi }\frac{2s\left(r+d\right)}{{\{{s}^{2}+{\left(r+d\right)}^{2}\}}^{2}} ,$$

11 $${H}_{s}=-\frac{\partial\Phi }{\partial s}=\frac{\gamma }{2\pi }\frac{{s}^{2}-\left(r+d\right)}{{\{{s}^{2}+{\left(r+d\right)}^{2}\}}^{2}} .$$

A direct variation of magnetic force is quantity of $H$, which is established by the following relation.

12 $$H=\sqrt{{{H}_{r}}^{2}+{{H}_{s}}^{2}}.$$

Temperature $T$ may be estimated linearly from magnetization $M$ as shown below.

$$M={K}_{1}(T-{T}_{\infty }),$$

Where ${K}_{1}$ is the ferromagnetic coefficient.

The following morphing catalysts are explored in order to understand the simplified form of flow steering equations,

$$u=\frac{as}{1-{\alpha }^{*}t}{f}^{\mathrm{^{\prime}}}\left(\eta \right), v=\frac{-R}{r+R}\sqrt{\frac{a\nu }{1-{\alpha }^{*}t}}f\left(\eta \right), \eta =\sqrt{\frac{a}{\nu (1-{\alpha }^{*}t)}}r, p=\frac{\rho {a}^{2}{s}^{2}}{{\left(1-{\alpha }^{*}t\right)}^{2}}P\left(\eta \right)$$

13 $$\kappa =\sqrt{\frac{a}{\nu (1-{\alpha }^{*}t)}}R, \theta =\frac{{T-T}_{\infty }}{{T}_{w}-{T}_{\infty }}, {C}_{a}={C}_{0}\phi \left(\eta \right), {C}_{b}={C}_{0}h(\eta ),$$

where the non-dimensional velocity, pressure, temperature, homogenic concentration, and heterogenic concentration regimes are listed in that order: ${f}^{\prime}\left(\eta \right), P\left(\eta \right), \theta (\eta )$, $\phi (\eta )$ and $h\left(\eta \right)$. In terms of the cohesive variable $\eta$, prime resembles differentiation; $\kappa$ is the curvature parameter and ${C}_{0}$ is the constant.

Expression (1) is identically verified and equations (2) to (6) become

14 $$\frac{\partial P}{\partial \eta }=\frac{{{f}^{\mathrm{^{\prime}}}}^{2}}{\eta +\kappa },$$

15 $$\frac{2\kappa }{\eta +\kappa }P=\frac{\kappa }{{\left(\eta +\kappa \right)}^{2}}f{f}^{\prime}+\frac{\kappa }{\eta +\kappa }f{f}^{{\prime}{\prime}}-\frac{\kappa }{\eta +\kappa }{{f}^{\prime}}^{2}+\left(1+\frac{1}{\beta }\right)\left[{f}^{{\prime}{\prime}{\prime}}+\frac{1}{(\eta +\kappa )}{f}^{{\prime}{\prime}}-\frac{1}{{\left(\eta +\kappa \right)}^{2}}{f}^{\prime}\right]+We\left[{f}^{{\prime}{\prime}}{f}^{{\prime}{\prime}{\prime}}+\frac{1}{(\eta +\kappa )}\left({{f}^{{\prime}{\prime}}}^{2}-{f}^{\prime}{f}^{{\prime}{\prime}{\prime}}\right)-\frac{2{f}^{\prime}{f}^{{\prime}{\prime}}}{{\left(\eta +\kappa \right)}^{2}}+\frac{{{f}^{\prime}}^{2}}{{\left(\eta +\kappa \right)}^{3}}\right]-{\delta }^{*}\left[{f}^{\prime}+\frac{\eta }{2}{f}^{{\prime}{\prime}}\right]-\frac{2{\beta }_{m}}{{\left(n+b\right)}^{4}}\theta +{\lambda }_{T}\theta -{M}^{*}{f}^{\prime},$$

16 $$\frac{1}{Pr}\left[1+Rd\right]\left(\frac{1}{\eta +\kappa }{\theta }^{\mathrm{^{\prime}}}+{\theta }^{\mathrm{^{\prime}}\mathrm{^{\prime}}}\right)+\frac{\kappa }{\eta +\kappa }f{\theta }^{\mathrm{^{\prime}}}+Nb{\phi }^{\mathrm{^{\prime}}}{\theta }^{\mathrm{^{\prime}}}+Nt{{\theta }^{\mathrm{^{\prime}}}}^{2}-\frac{\eta }{2}{\delta }^{*}{\theta }^{\mathrm{^{\prime}}}+Ec {M}^{*}{{f}^{\mathrm{^{\prime}}}}^{2}+Q\theta {e}^{-\eta }-{C}_{H}{\left(\frac{\kappa }{\eta +\kappa }\right)}^{2}\left[{f}^{2}{\theta }^{\mathrm{^{\prime}}\mathrm{^{\prime}}}+f{f}^{\mathrm{^{\prime}}}{\theta }^{\mathrm{^{\prime}}}-\frac{{f}^{2}}{\eta +\kappa }{\theta }^{\mathrm{^{\prime}}}\right]+\frac{2}{Pr}\frac{{\beta }_{m}{\lambda }_{m}\left(\theta -\epsilon \right)}{{\left(\eta +b\right)}^{3}}\left[\frac{\kappa f}{\eta +\kappa }\left\{1-\frac{2}{{\left(\eta +b\right)}^{2}}\right\}-\frac{{f}^{\mathrm{^{\prime}}}}{\eta +b}\right]=0,$$

17 $$\frac{1}{Sc}\left(\frac{{\phi }^{\mathrm{^{\prime}}}}{\eta +\kappa }+{\phi }^{\mathrm{^{\prime}}\mathrm{^{\prime}}}\right)+\frac{\delta }{Sc}\frac{Nt}{Nb}\left({\theta }^{\mathrm{^{\prime}}\mathrm{^{\prime}}}+\frac{1}{\eta +\kappa }{\theta }^{\mathrm{^{\prime}}}\right)+\frac{\kappa }{\eta +\kappa }f{\phi }^{\mathrm{^{\prime}}}-\frac{\eta }{2}{\delta }^{*}{\phi }^{\mathrm{^{\prime}}}-{k}_{1}\phi {h}^{2}=0,$$

18 $$\frac{\delta }{Sc}\left(\frac{{h}^{\mathrm{^{\prime}}}}{\eta +\kappa }+{h}^{\mathrm{^{\prime}}\mathrm{^{\prime}}}\right)+\frac{\delta }{Sc}\frac{Nt}{Nb}\left({\theta }^{\mathrm{^{\prime}}\mathrm{^{\prime}}}+\frac{1}{\eta +\kappa }{\theta }^{\mathrm{^{\prime}}}\right)+\frac{\kappa }{\eta +\kappa }f{h}^{\mathrm{^{\prime}}}-\frac{\eta }{2}{\delta }^{*}{h}^{\mathrm{^{\prime}}}+{k}_{1}\phi {h}^{2}=0.$$

The following are the transfused boundary conditions:

19 $${f}^{\mathrm{{\prime}}}=1+{L}_{1}\left({f}^{\mathrm{{\prime}}\mathrm{{\prime}}}-\frac{1}{\kappa }{f}^{\mathrm{{\prime}}}\right), Me{\theta }^{\mathrm{{\prime}}}+Prf=0, \theta =1, {\phi }^{\mathrm{{\prime}}}={k}_{2}\phi , \delta {h}^{\mathrm{{\prime}}}=-{k}_{2}\phi \,\,\mathrm{ at }\,\,\eta =0, {f}^{\prime}\to 0, {f}^{{\prime}{\prime}}\to 0, \theta \to 0 , \phi \to 1\,\,\mathrm{ as }\,\,\eta \to \infty ,$$

where $We\left(=\sqrt{\frac{{2a}^{3}}{\nu {\left(1-{\alpha }^{*}t\right)}^{3}}}\Gamma s\right)$-local Weissenberg number, ${\delta }^{*}\left(=\frac{{\alpha }^{*}}{a}\right)$-unsteadiness parameter, ${\beta }_{m}\left(=\frac{\gamma {\mu }_{0}{K}_{1}\left({T}_{w}-{T}_{\infty }\right)\rho }{2\pi {\mu }^{2}}\right)$-ferrohydrodynamic interaction, $b\left(=\sqrt{\frac{a}{\nu (1-{\alpha }^{*}t)}}d\right)$-dimensionless distance, ${\lambda }_{T}\left(=\frac{Gr}{R{e}^{2}}\right)$-thermal Buoyancy Parameter, $Gr\left(=\frac{g{\beta }_{T}{T}_{\infty }\left({\theta }_{w}-1\right){s}^{3}}{{\nu }^{2}}\right)$ corresponds to the local Grashof number, ${M}^{*}\left(=\frac{\sigma {B}_{0}^{2}}{\rho a}\right)$-magnetic parameter, $\mathrm{Pr}\left(=\frac{\nu }{\alpha }\right)$-Prandtl number, $Rd\left(=\frac{16{\sigma }^{*}{T}_{\infty }^{3}}{3k{k}^{*}}\right)$-radiation parameter, $Nt\left(=\frac{\tau {D}_{T}\left({T}_{w}-{T}_{\infty }\right)}{\nu {T}_{\infty }}\right)$-thermophoresis parameter, $Nb\left(=\frac{\tau {D}_{{C}_{b}}{C}_{0}}{\nu }\right)$-Brownian motion parameter, $Ec\left(=\frac{{u}_{w}^{2}}{{c}_{p}\left({T}_{w}-{T}_{\infty }\right)}\right)$-Eckert number, ${C}_{H}\left(={\lambda }_{1}{a}_{1}\right)$-thermal relaxation parameter, ${\lambda }_{m}\left(=\frac{a{\mu }^{2}}{\rho k({T}_{w}-{T}_{\infty } )(1-{\alpha }^{*}t)}\right)$-heat dissipation parameter, $\epsilon \left(=\frac{{T}_{\infty }}{{T}_{\infty }-{T}_{w}}\right)$-Curie temperature, $Q\left(=\frac{{Q}_{0}(1-{\alpha }^{*}t)}{a\left(\rho {c}_{p}\right)}\right)$-heat source/sink parameter, $Sc\left(=\frac{\nu }{{{D}_{C}}_{a}}\right)$-Schmidt number, $\delta \left(=\frac{{D}_{{C}_{b}}}{{D}_{{C}_{a}}}\right)$-ratio of diffusion coefficients, ${k}_{1}\left(=\frac{{C}_{0}^{2}{k}_{c}(1-{\alpha }^{*}t)}{a}\right)$-homogenic reaction parameter, ${k}_{2}\left(=\frac{{k}_{s}}{{D}_{{C}_{a}}}\sqrt{\frac{\nu (1-{\alpha }^{*}t)}{a}}\right)$-heterogenic reaction parameter, ${L}_{1}=\left(Ls\sqrt{\frac{a}{\nu (1-{\alpha }^{*}t)}}\right)$-slip parameter and $Me\left(=\frac{{c}_{p}\left({T}_{w}-{T}_{\infty }\right)}{{\lambda }_{l}+\left({T}_{w}-{T}_{0}\right){c}_{s}}\right)$-melting heat parameter.

When pressure $P(\eta )$ is removed from Eqs. (14) and (15), it results in.

20 $$\left(1+\frac{1}{\beta }\right)\left[{f}^{\mathrm{^{\prime}}v}+\frac{2{f}^{\mathrm{^{\prime}}\mathrm{^{\prime}}\mathrm{^{\prime}}}}{\eta +\kappa }-\frac{{f}^{\mathrm{^{\prime}}\mathrm{^{\prime}}}}{{\left(\eta +\kappa \right)}^{2}}+\frac{{f}^{\mathrm{^{\prime}}}}{{\left(\eta +\kappa \right)}^{3}}\right]+We\left[\left({f}^{\mathrm{^{\prime}}\mathrm{^{\prime}}}{f}^{\mathrm{^{\prime}}v}+{{f}^{\mathrm{^{\prime}}\mathrm{^{\prime}}\mathrm{^{\prime}}}}^{2}\right)-\frac{1}{\eta +\kappa }\left({f}^{\mathrm{^{\prime}}}{f}^{\mathrm{^{\prime}}v}-2{f}^{\mathrm{^{\prime}}\mathrm{^{\prime}}}{f}^{\mathrm{^{\prime}}\mathrm{^{\prime}}\mathrm{^{\prime}}}\right)-\frac{2}{{\left(\eta +\kappa \right)}^{2}}\left({{f}^{\mathrm{^{\prime}}\mathrm{^{\prime}}}}^{2}+{f}^{\mathrm{^{\prime}}}{f}^{\mathrm{^{\prime}}\mathrm{^{\prime}}\mathrm{^{\prime}}}\right)+\frac{4{f}^{\mathrm{^{\prime}}}{f}^{\mathrm{^{\prime}}\mathrm{^{\prime}}}}{{\left(\eta +\kappa \right)}^{3}}-\frac{2{{f}^{\mathrm{^{\prime}}}}^{2}}{{\left(\eta +\kappa \right)}^{4}}\right]+\frac{\kappa }{\eta +\kappa }\left[f{f}^{\mathrm{^{\prime}}\mathrm{^{\prime}}\mathrm{^{\prime}}}-{f}^{\mathrm{^{\prime}}}{f}^{\mathrm{^{\prime}}\mathrm{^{\prime}}}\right]+\frac{\kappa }{{\left(\eta +\kappa \right)}^{2}}\left[f{f}^{\mathrm{^{\prime}}\mathrm{^{\prime}}}-{{f}^{\mathrm{^{\prime}}}}^{2}\right] -\frac{\kappa }{{\left(\eta +\kappa \right)}^{3}}f{f}^{\mathrm{^{\prime}}}-{\delta }^{*}\left[\frac{\eta }{2}{f}^{\mathrm{^{\prime}}\mathrm{^{\prime}}\mathrm{^{\prime}}}+\frac{3{f}^{\mathrm{^{\prime}}\mathrm{^{\prime}}}}{2}\right]-\frac{{\delta }^{*}}{\eta +\kappa }\left[\frac{\eta }{2}{f}^{\mathrm{^{\prime}}\mathrm{^{\prime}}}+{f}^{\mathrm{^{\prime}}}\right]-\frac{2{\beta }_{m} }{{\left(\eta +b\right)}^{4}}\left[\left(\frac{1}{\eta +\kappa }-\frac{4}{\eta +b}\right)\theta +{\theta }^{\mathrm{^{\prime}}}\right]+{\lambda }_{T}\left[\frac{\theta }{\eta +\kappa }+{\theta }^{\mathrm{^{\prime}}}\right]-{M}^{*}\left[{f}^{\mathrm{^{\prime}}\mathrm{^{\prime}}}+\frac{{f}^{\mathrm{^{\prime}}}}{\eta +\kappa }\right]=0.$$

When ${D}_{{C}_{a}}={D}_{{C}_{b}}$, $\delta =1$ and $\phi \left(\eta \right)+h\left(\eta \right)=1$ are both true.

Now equations (17) and (18) become

21 $$\frac{1}{Sc}\left(\frac{{\phi }^{\prime}}{\eta +\kappa }+{\phi }^{{\prime}{\prime}}\right)+\frac{\kappa }{\eta +\kappa }f{\phi }^{\prime}-{k}_{1}\phi {\left(1-\phi \right)}^{2}-\eta {\delta }^{*}{\phi }^{\prime}=0,$$

with boundary conditions.

22 $$\phi^{\prime}\left( 0 \right) = k_{2} \phi \left( 0 \right), \phi \left( \infty \right) \to 1.$$

The following skin friction coefficient and the Nusselt number, are characteristics of engineering prominence.

23 $${Cf}_{s}=\frac{{\tau }_{w}}{\rho {u}_{w}^{2}} ,$$

24 $$N{u}_{s}=\frac{s{q}_{w}}{\left({T}_{w}-{T}_{\infty }\right)k}$$

here ${\tau }_{w}$-wall shear stress and ${q}_{w}$-wall heat flux which are given by.

25 $${\tau }_{w}={\left.\mu \left[\left(1+\frac{1}{\beta }\right)\left(\frac{\partial u}{\partial r}+\frac{R}{R+r}\frac{\partial v}{\partial s} -\frac{u}{r+R}\right)+\frac{{\Gamma }^{2}}{\sqrt{2}}{\left(\frac{\partial u}{\partial r}+\frac{R}{R+r}\frac{\partial v}{\partial s} -\frac{u}{r+R}\right)}^{2}\right]\right|}_{r=0},$$

26 $${q}_{w}={\left.-k\left(\frac{\partial T}{\partial r}\right)\right|}_{r=0}+{\left.{q}_{r}\right|}_{r=0}.$$

Reduced form of above is

27 $$C{f}_{s}{(Re)}^\frac{1}{2}=\left(1+\frac{1}{\beta }\right)\left[{f}^{\mathrm{^{\prime}}\mathrm{^{\prime}}}\left(0\right)-\frac{1}{\kappa }{f}^{\mathrm{^{\prime}}}\left(0\right)\right]+\frac{We}{2}{\left[{f}^{\mathrm{^{\prime}}\mathrm{^{\prime}}}\left(0\right)-\frac{1}{\kappa }{f}^{\mathrm{^{\prime}}}\left(0\right)\right]}^{2},$$

28 $$N{u}_{s}{(Re)}^{-\frac{1}{2}}={-\left[1+Rd\right]\theta }^{\prime}\left(0\right),$$

where $Re=\frac{a{s}^{2}}{\nu }$ denotes local Reynolds number.

## Numerical procedure

It is possible to ensure the accuracy of the IVP solution by repeating the simplified equations twice with step lengths of $h$ and $h/2.$

To establish good synergy, this process must first undergo extensive simulation due to the shorter step length. One of these methods, the Runge–Kutta Fehlberg scheme, contains a protocol to determine whether the appropriate step length is being used. Every step yields two accurate approximations of the solution, which are then discussed. If the two answers closely synergize, the accuracy of the approximation is impaired; otherwise, the step size is reduced. If the answer settles on more digits, the step length is adjusted. Each step results in values as below:

$${k}_{1}=hf\left({x}_{i},{y}_{i}\right),$$

$${k}_{2}=hf\left({x}_{i}+\frac{h}{24},{y}_{i}+\frac{{k}_{1}}{4}\right),$$

$${k}_{3}=hf\left({x}_{i}+\frac{3h}{8},{y}_{i}+\frac{3{k}_{1}}{32}+\frac{9{k}_{2}}{32}\right), -11k.$$

$${k}_{4}=hf\left({x}_{i}+\frac{12h}{13},{y}_{i}+\frac{1932{k}_{1}}{2197}-\frac{7200{k}_{2}}{2197}+\frac{7296{k}_{3}}{2197}\right),$$

$${k}_{5}=hf\left({x}_{i}+h,{y}_{i}+\frac{439{k}_{1}}{216}-8{k}_{2}+\frac{3680{k}_{3}}{513}-\frac{845{k}_{4}}{4104}\right),$$

$${k}_{6}=hf\left({x}_{i}+\frac{h}{2},{y}_{i}+\frac{8}{27}{k}_{1}+2{k}_{2}-\frac{3544{k}_{3}}{2565}+\frac{1859{k}_{4}}{4104}-\frac{11{k}_{5}}{40}\right).$$

Then an approximation using 4^th^order RK-method is.

$${y}_{i+1}={y}_{i}+\frac{25{k}_{1}}{216}+\frac{1408{k}_{3}}{2565}+\frac{2197{k}_{4}}{4104}-\frac{1{k}_{5}}{5}.$$

It is noteworthy that ${k}_{2}$ value is not counted in the above given formula. The other value of y is known by 5^th^order RK-method as:

$${y}_{i+1}^{*}={y}_{i}+\frac{16{k}_{1}}{135}+\frac{6656{k}_{3}}{12825}+\frac{28561{k}_{4}}{56430}-\frac{9{k}_{5}}{50}+\frac{2{k}_{6}}{55}.$$

If $\left|{y}_{i+1}+{y}_{i+1}^{*}\right|$ is small enough, then the method is terminated; or else the simulation is carried on using lesser step size $h$. The local truncation error is ${y}_{i+1}-{y}_{\left(i+1\right)}^{*}$.

## Results and their deliberation

A continuously elongated magnetic dipole-enabled curved melting surface equipped with a magnetic field, Joule heating, an exponential space-based heat source, thermal radiation, and homo-heterogenic reactions has been envisioned mathematically, with special attention paid to the Cattaneo-Christov heat flux model and buoyancy effect. The slip condition is enhanced at the melting surface boundary. The use of similarity catalysts converts the preset equations into straightforward ordinary differential equations. The examined flow issue is represented graphically using the Runge–Kutta–Fehlberg 4-5^th^ order approach, an effective numerical tool. The rest of the parameter values have been preserved at their usual values as $\beta =2, \kappa =4, We=0.1, {M}^{*}=1, {\lambda }_{T}=2.5, {C}_{H}=0.3, {\delta }^{*}=0.1, {\beta }_{m}=1, b=1, Ec=0.1, Rd=1.5, Pr=7, Nt=0.01, Nb=0.01, Q=0.1, {\lambda }_{m}=0.5, \epsilon =0.5, Sc=7, {k}_{1}=0.1, {k}_{2}=0.1, {L}_{1}=0.1, Me=0.7$ while carrying out numerical extractions for all flow fields against the relevant parameters. Table 1 compares the bvp4c methodology developed by Zhang et al.^42^ with the present numerical method to provide validation. There appears to be a reasonable amount of consistency among the data in the table. Significantly more detail has been provided on each resulting graph.

**Table 1:** Comparison of ${f}^{{\prime}{\prime}}\left(0\right)-\frac{{f}^{\prime}\left(0\right)}{\kappa }$ between present study and previous study^42^.

$\kappa$	Zhang et al.^41^	Present results
5	1.15763	1.15763
10	1.07349	1.07349
20	1.03561	1.03561
30	1.02353	1.02353
40	1.01759	1.01759
50	1.01405	1.01405

**Figure 2:**
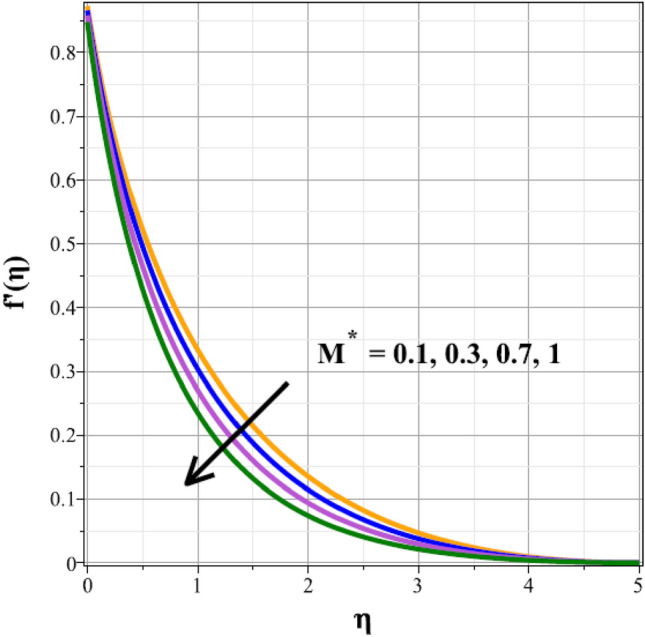
Curves of $f^{\prime}(\eta )$ for ${M}^{*}$.

**Figure 3:**
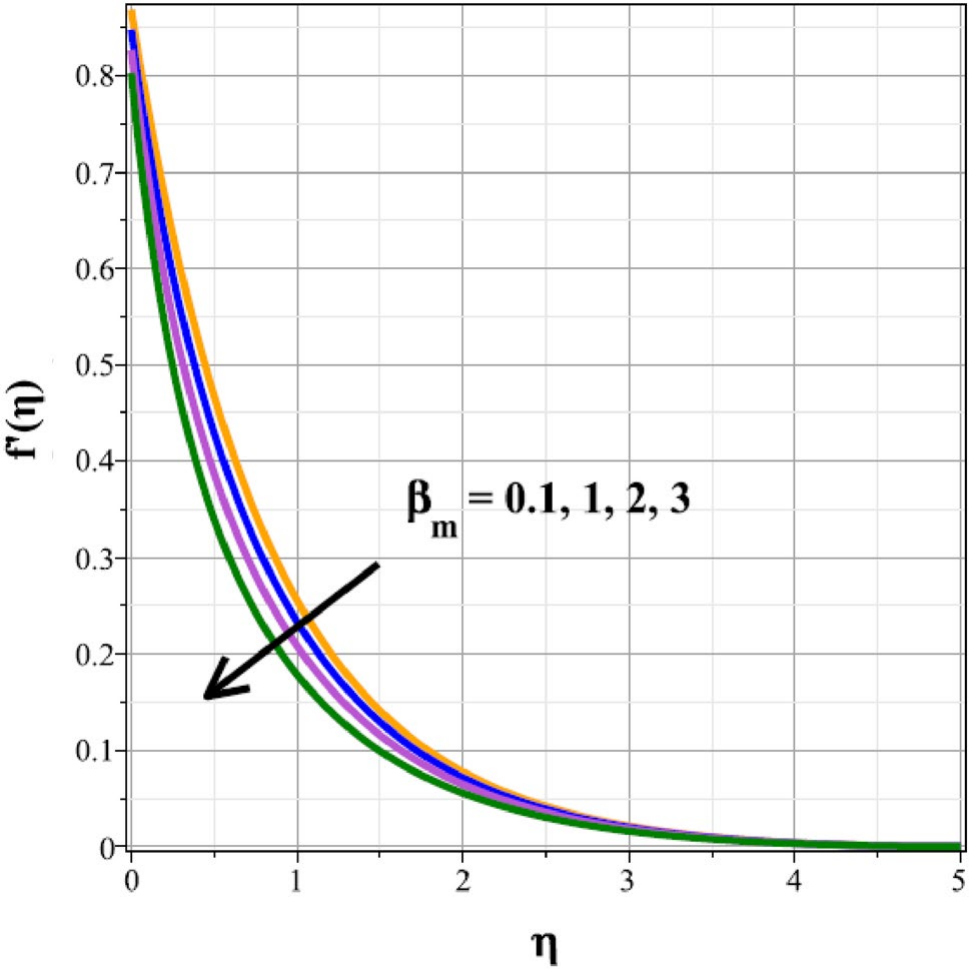
Curves of ${f}^{\prime}(\eta )$ for ${\beta }_{m}$.

**Figure 4:**
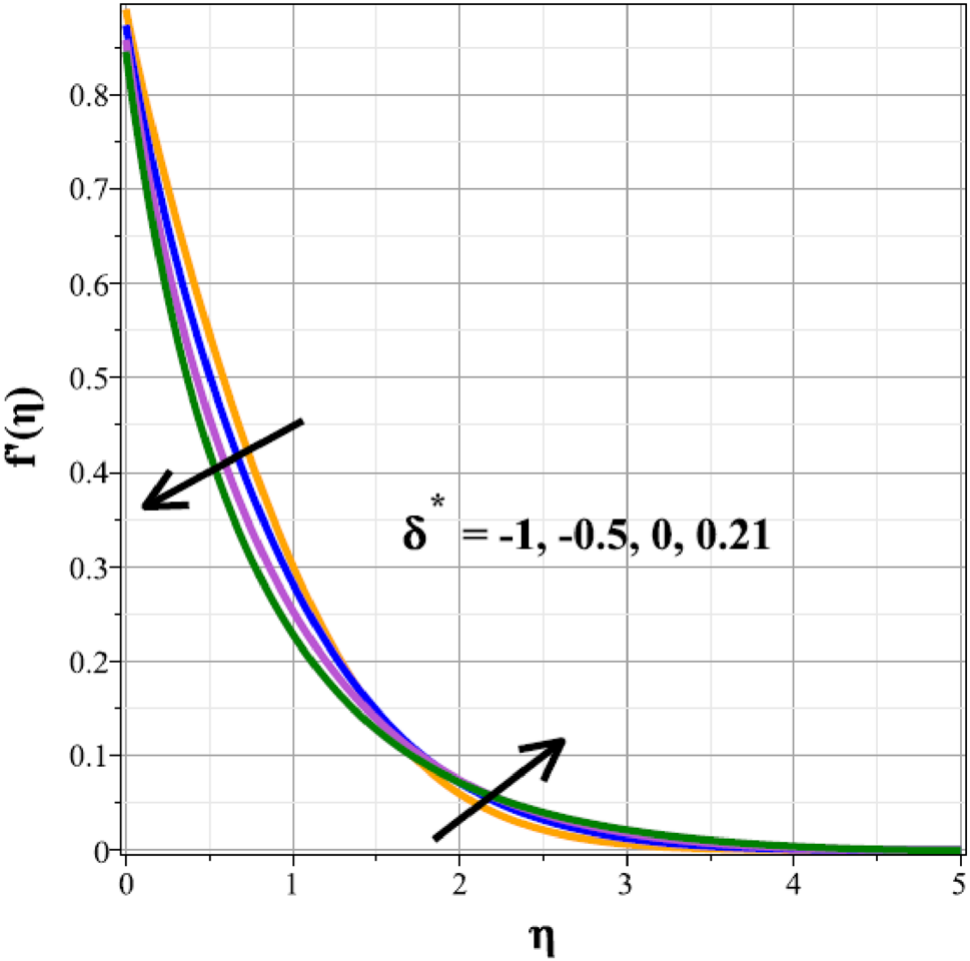
Curves of $f^{\prime}(\eta )$ for ${\delta }^{*}$.

**Figure 5:**
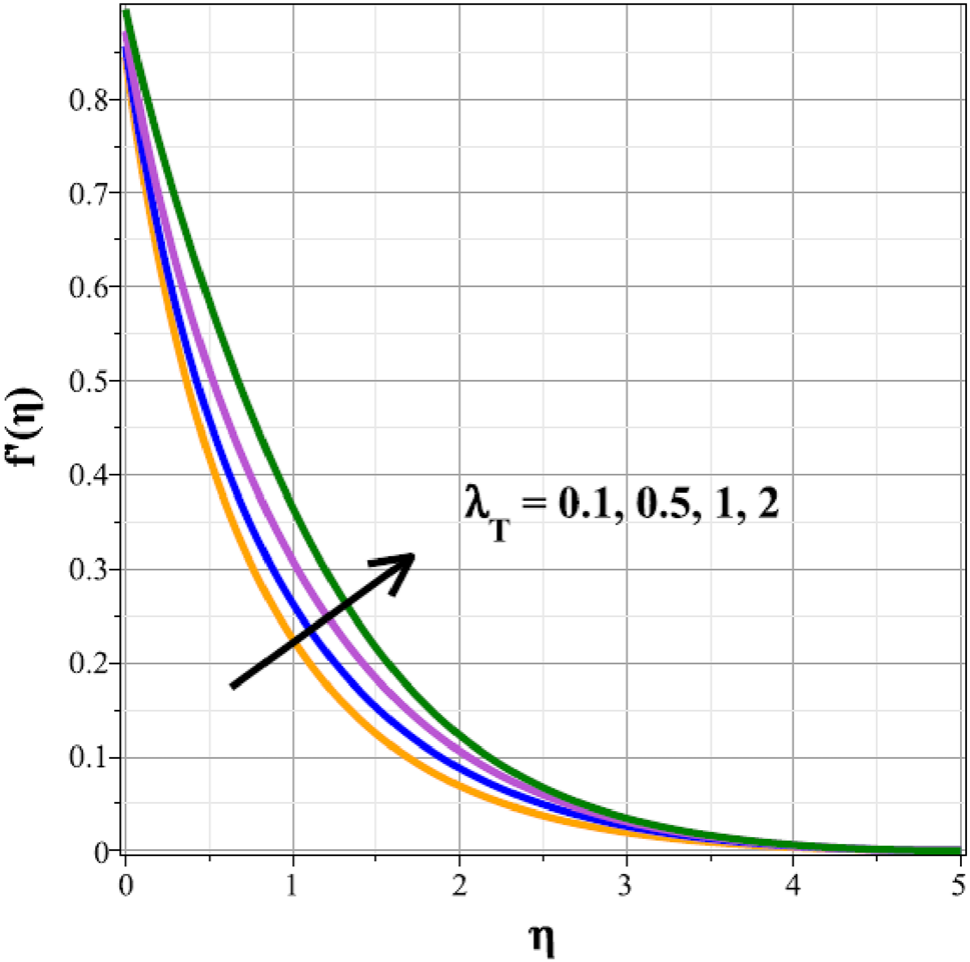
Curves of ${f}^{\prime}(\eta )$ for ${\lambda }_{T}$.

**Figure 6:**
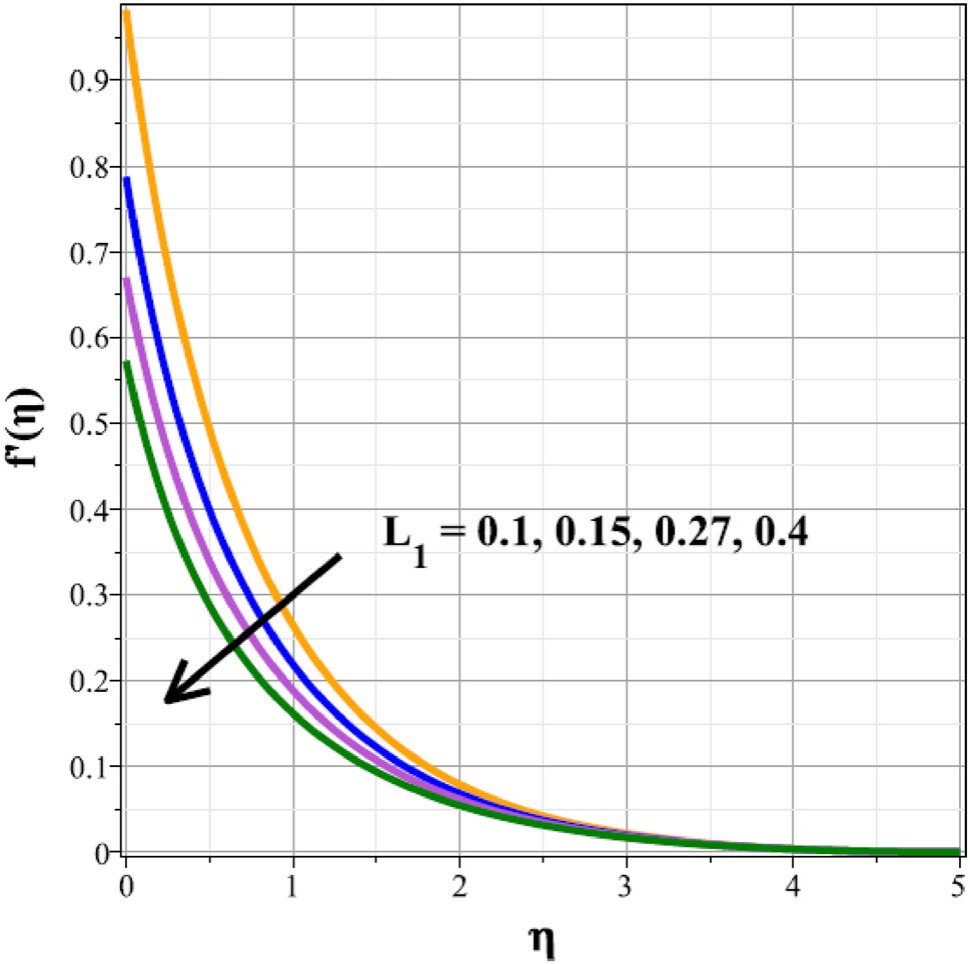
Curves of $f^{\prime}(\eta )$ for ${L}_{1}$.

**Figure 7:**
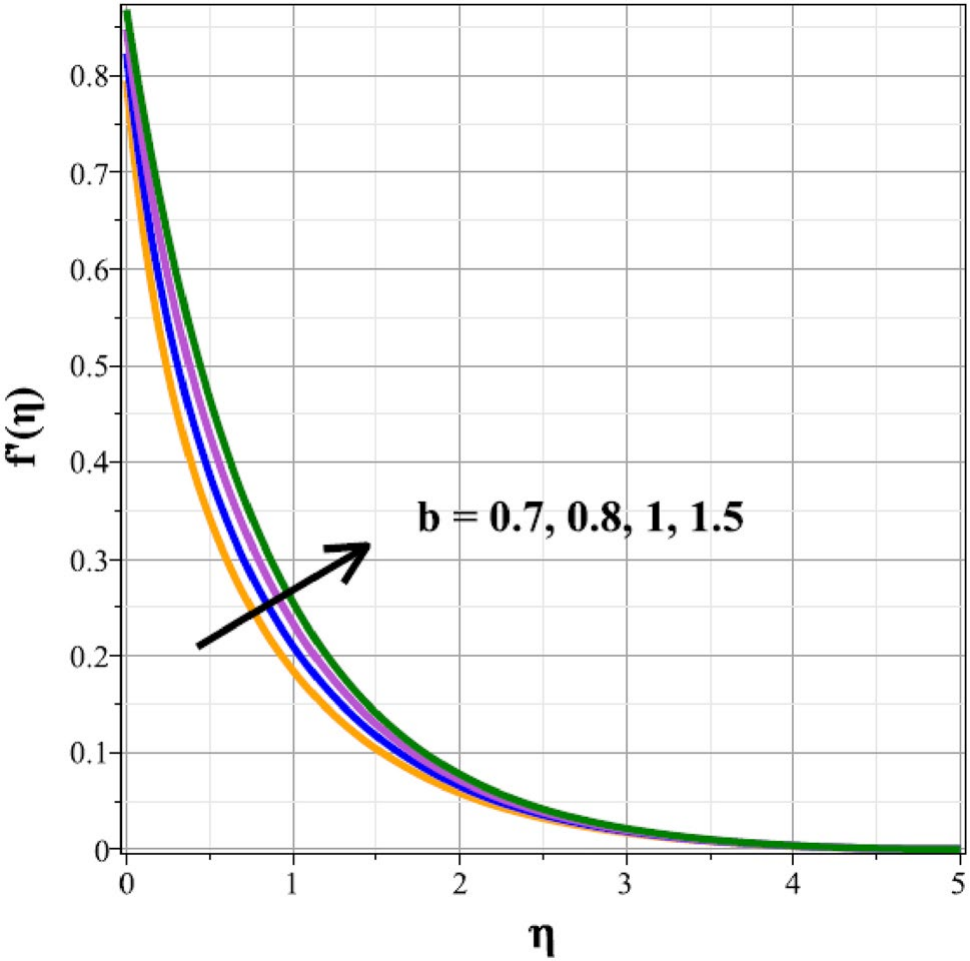
Curves of ${f}^{\prime}(\eta )$ for $b$.

Plots of the behavioral changes in the velocity panel $(f^{\prime}(\eta ))$ for the magnetic parameter $({M}^{*})$, ferrohydrodynamic interaction parameter $({\beta }_{m})$, unsteady factor $({\delta }^{*})$, thermal buoyancy factor $({\lambda }_{T})$, velocity slip factor $({L}_{1})$ and dipole distance $(b)$ are shown in Figs. 2, 3, 4, 5, 6 and 7, respectively. The variations in velocity ($f^{\prime}(\eta )$) for ascending magnetic attribute (${M}^{*}$) values are clearly shown in Fig. 2, which is declining in nature. The Lorentz force, which is present and amplified by the increase in ${M}^{*}$, is the cause of this velocity impedance. When increasing values of ${\beta }_{m}$ are present, it is clear from Fig. 3 that the velocity panel de-escalates. The velocity distribution is decreased as a result of the dominance of the ferrohydrodynamic interaction factor and increased Lorentz force. The fluid's behavior increases as it moves farther from the sheet, as seen by the indicated behavior of the velocity distribution in Fig. 4 for expanding values of ${\delta }^{*}$. The explanation for this is because a rise in ${\delta }^{*}$ causes an increase in the reciprocity of time factor, which slows the pace at which the sheet stretches. The velocity regime for the parameter ${\lambda }_{T}$ is shown in Fig. 5. The relative variable impact of the thermal floating force on the flow of nanofluid is personified by it. As seen in Fig. 5, the increase in thermal buoyancy force causes the flow to move more quickly. Figure 6 clearly illustrates the influence of the velocity slip factor's de-escalation on the velocity distribution. In stretched sheets and fluid flows, an increase in ${L}_{1}$ creates heterogenic velocity, which results in a decrease in the velocity distribution. The effect of a magnetic dipole's dimensionless distance on the velocity regime is briefly explored in Fig. 7. The velocity regime is shown to steadily grow as $b$ increases, despite the dipole distance increasing.

**Figure 8:**
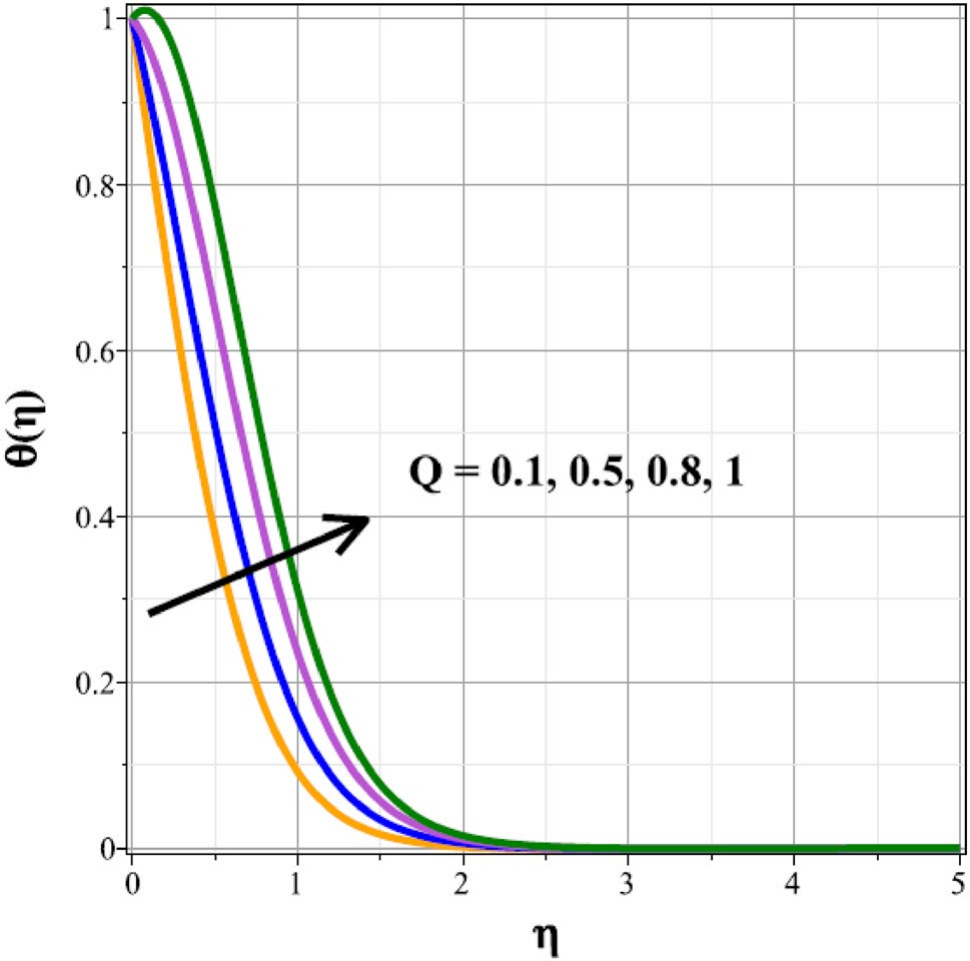
Curves of $\theta (\eta )$ for $Q$.

**Figure 9:**
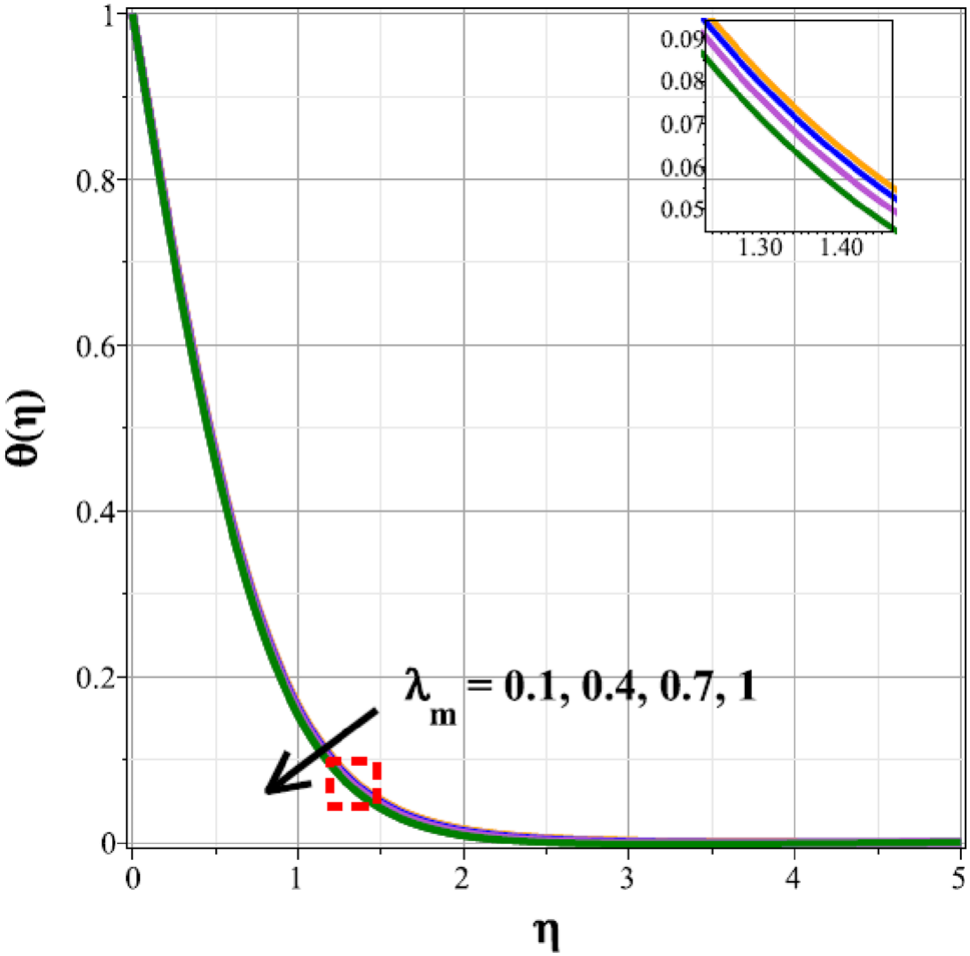
Curves of $\theta (\eta )$ for ${\lambda }_{m}$.

**Figure 10:**
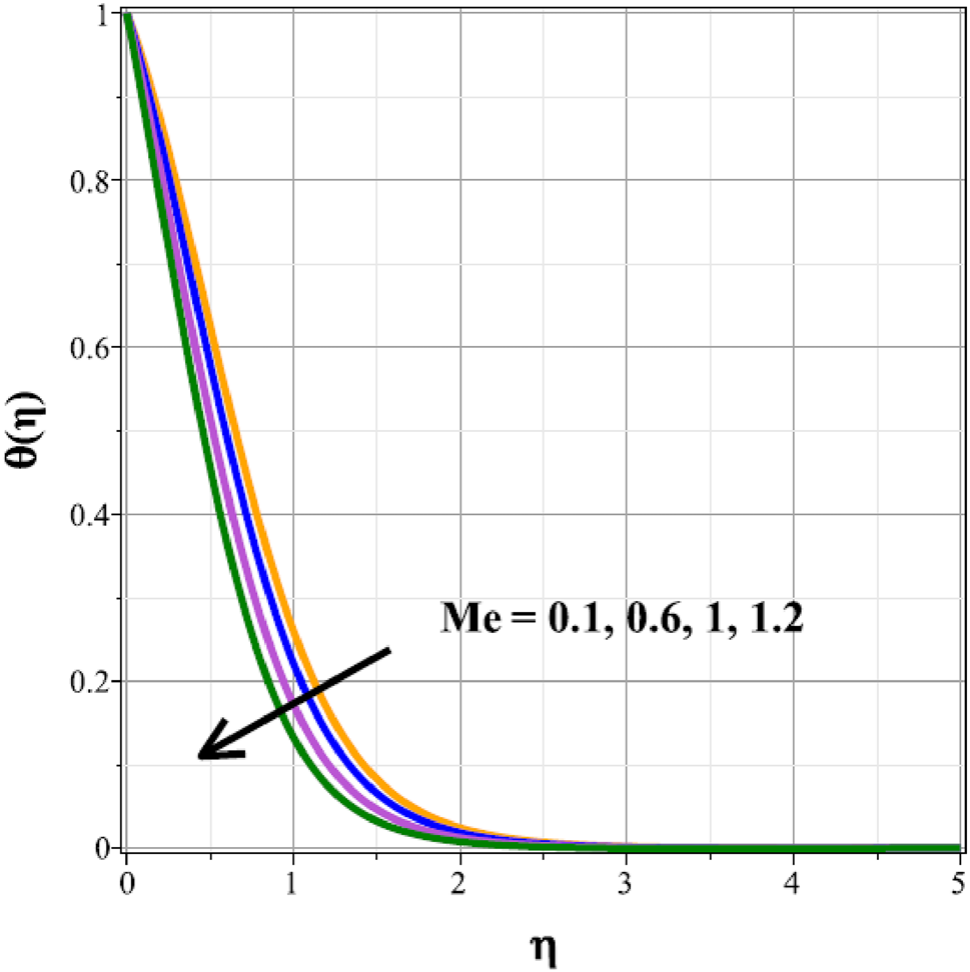
Curves of $\theta (\eta )$ for $Me$.

**Figure 11:**
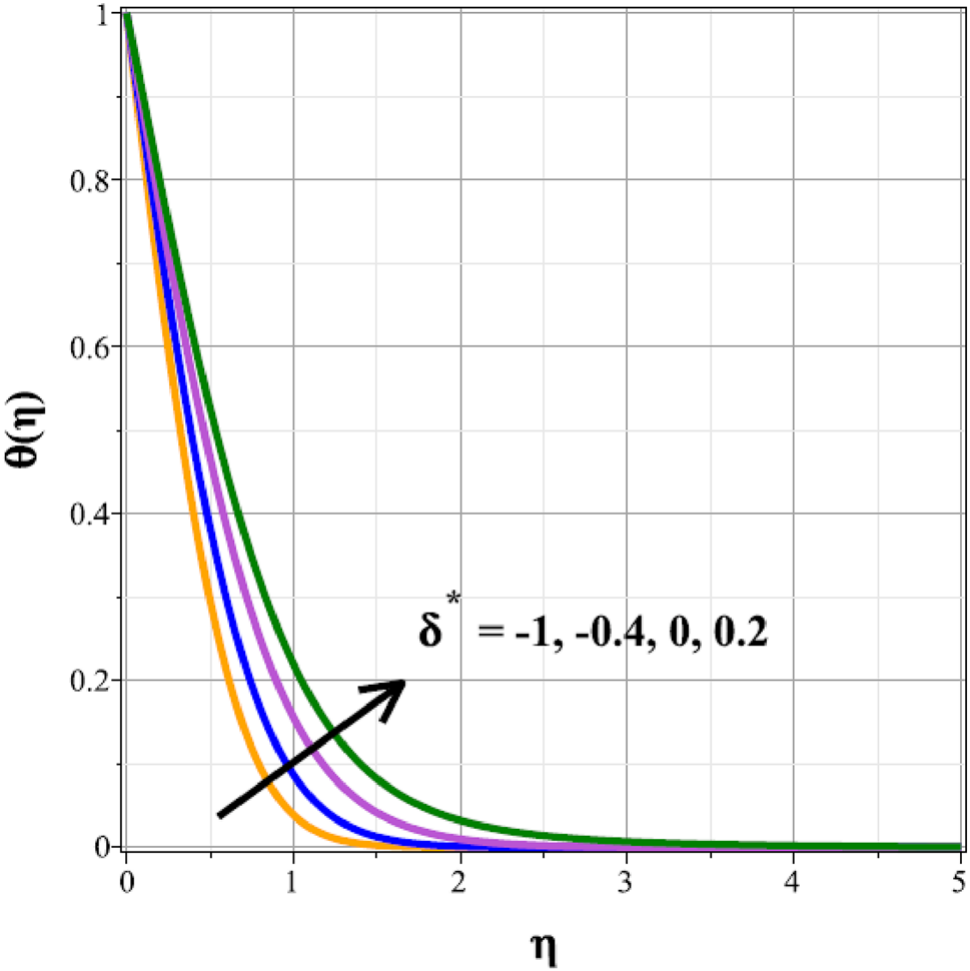
Curves of $\theta (\eta )$ for ${\delta }^{*}$.

**Figure 12:**
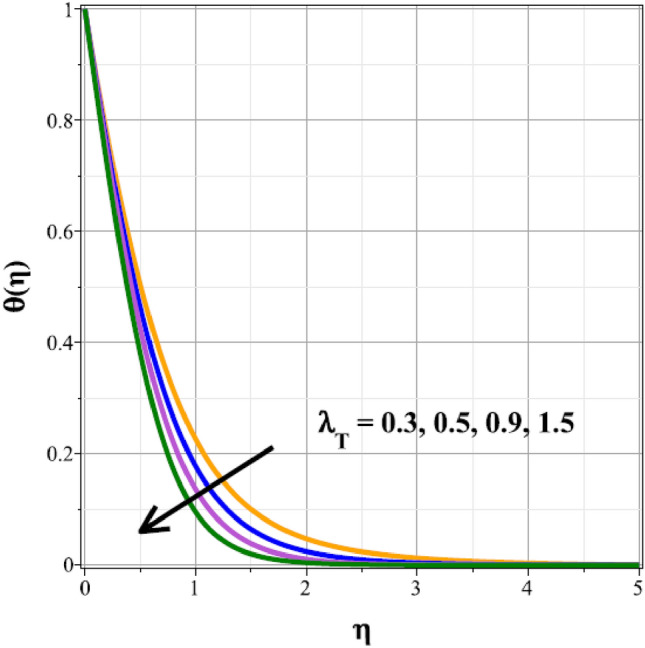
Curves of $\theta (\eta )$ for ${\lambda }_{T}$.

**Figure 13:**
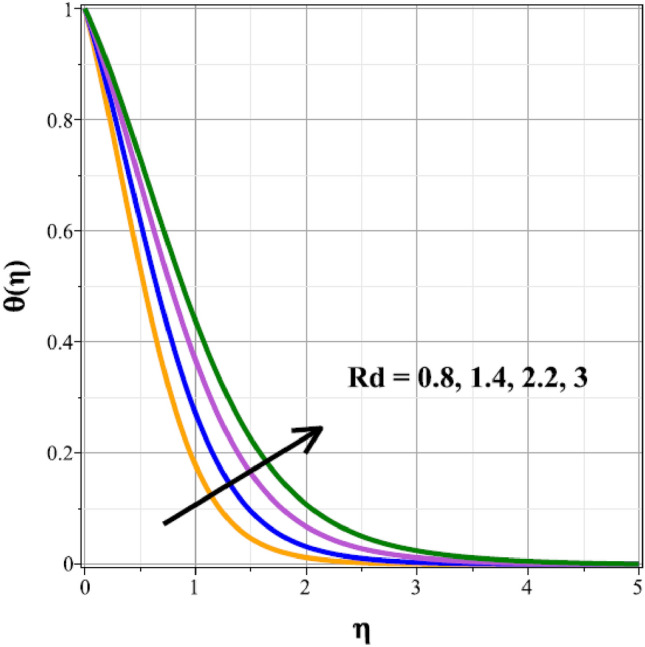
Curves of $\theta (\eta )$ for $Rd$.

**Figure 14:**
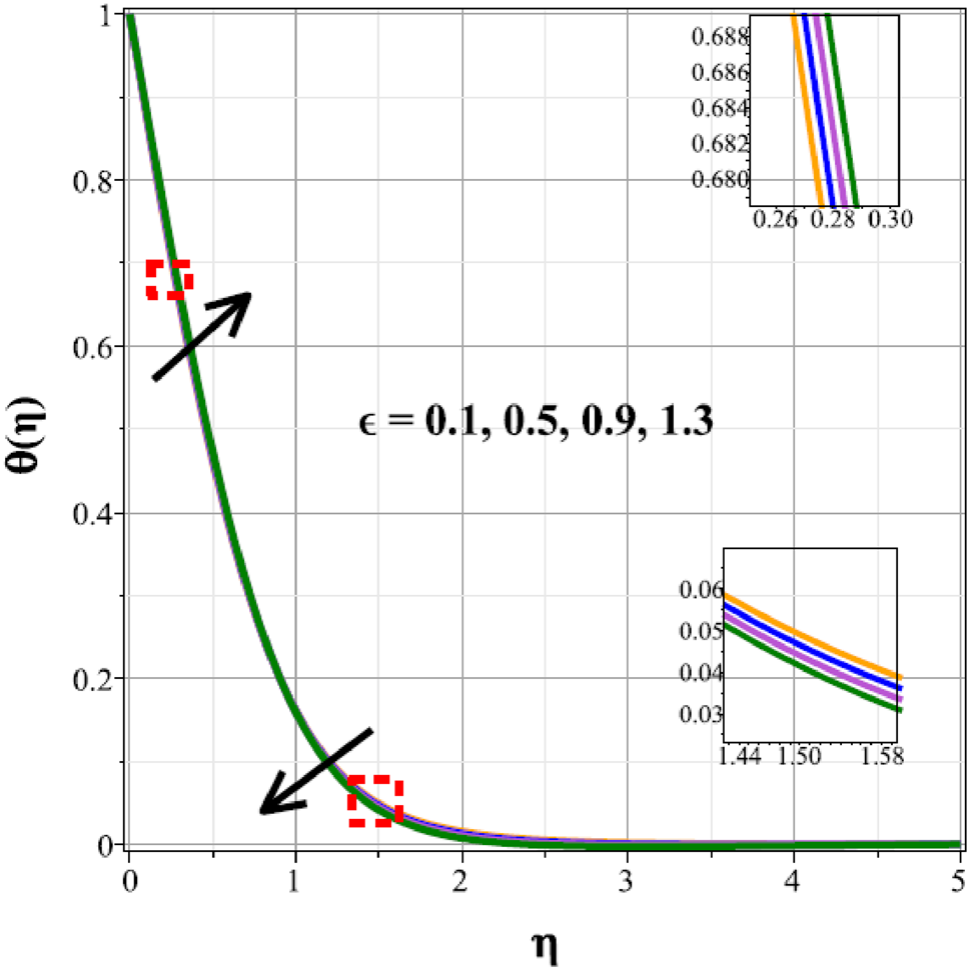
Curves of $\theta (\eta )$ for $\epsilon$.

**Figure 15:**
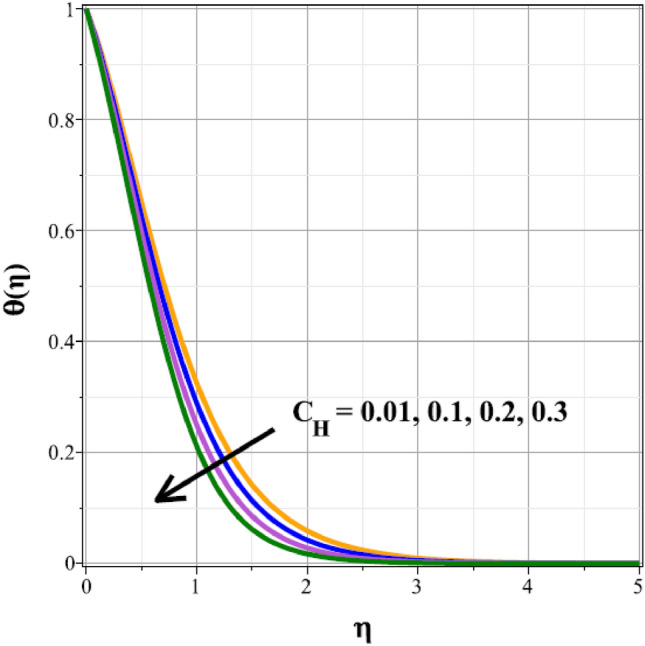
Curves of $\theta (\eta )$ for ${C}_{H}$.

The fluctuations of the thermal regime are shown in Figs. 8, 9, 10, 11, 12, 13, 14 and 15 for the components of exponential heat generation $(Q)$, heat dissipation $({\lambda }_{m})$, melting heat $(Me)$, unsteadiness $({\delta }^{*})$, thermal buoyancy $({\lambda }_{T})$, radiation $(Rd)$, Curie temperature $(\epsilon )$ and thermal relaxation $({C}_{H})$ in sequential order. Figure 8 shows the changes of the thermal panel for changing $Q$, and it thrives as a result of the heat creation within the nanofluid flow. Temperature panel changes in response to rising heat dissipation factor values. The temperature panel reduces as ${\lambda }_{m}$ grows due to an increase in the transfer of heat away from the sheet, as seen in Fig. 9. Because of the combined effects of radiation and the melting heat phenomena, which is depicted in Fig. 10, the thermal regime decreases with increasing values of $Me$. Figure 11 depicts the growing behaviour of the thermal distribution for expanding values of ${\delta }^{*}$ as a result of the cyclical behaviour of the stretching sheet. For rising levels of ${\lambda }_{T}$, Fig. 12 shows the thermal regime's decrementing characteristic. Because there are more thermal floating forces present when the temperature is raised, the hotness of the fluid is replaced by coolness, which causes the thermal panel to sink. Figure 13 shows how the radiation parameter $(Rd)$ affects the temperature panel. Because of the decrease in mean absorption coefficient, the thermal profile improves at higher values of $Rd$. Figure 14 shows the thermal regime curves with rising Curie temperature values. The thermal regime thrives as the value of it is raised because it raises the ambient temperature close to the sheet, while the thermal regime deteriorates for the fluid farther from the sheet. Figure 15 depicts how the temperature regime changes as ${C}_{H}$ values increase. The reason for this is thought to be due to the thermal boundary layer eroding.

**Figure 16:**
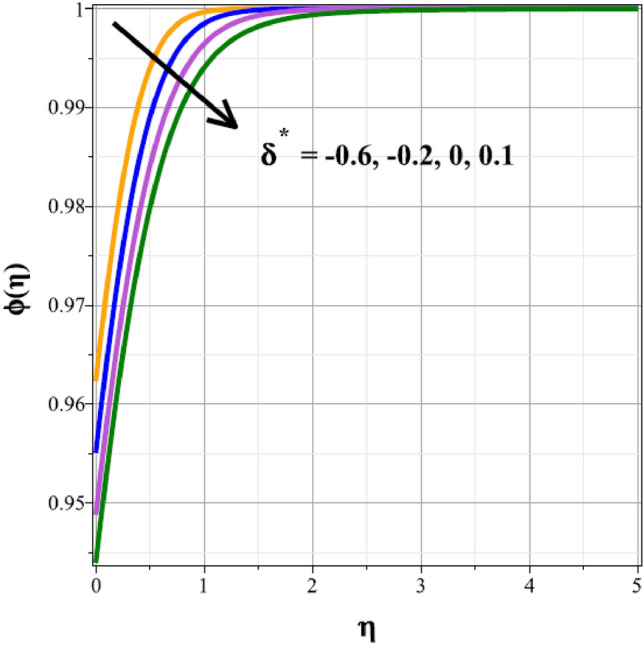
Curves of $\phi (\eta )$ for ${\delta }^{*}$.

**Figure 17:**
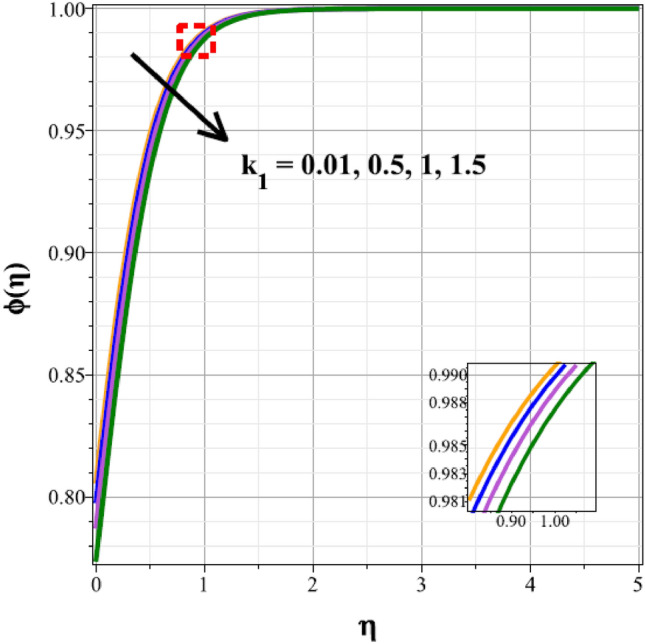
Curves of $\phi (\eta )$ for ${k}_{1}$.

**Figure 18:**
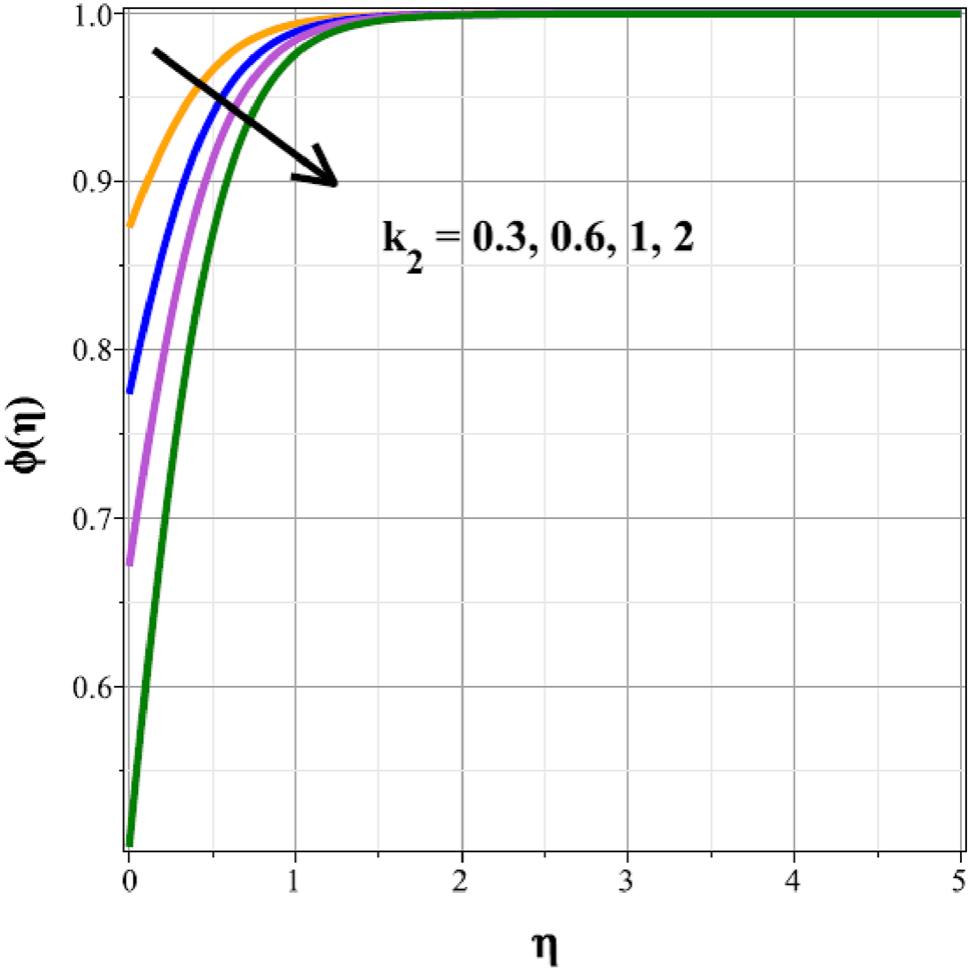
Curves of $\phi (\eta )$ for ${k}_{2}$.

**Figure 19:**
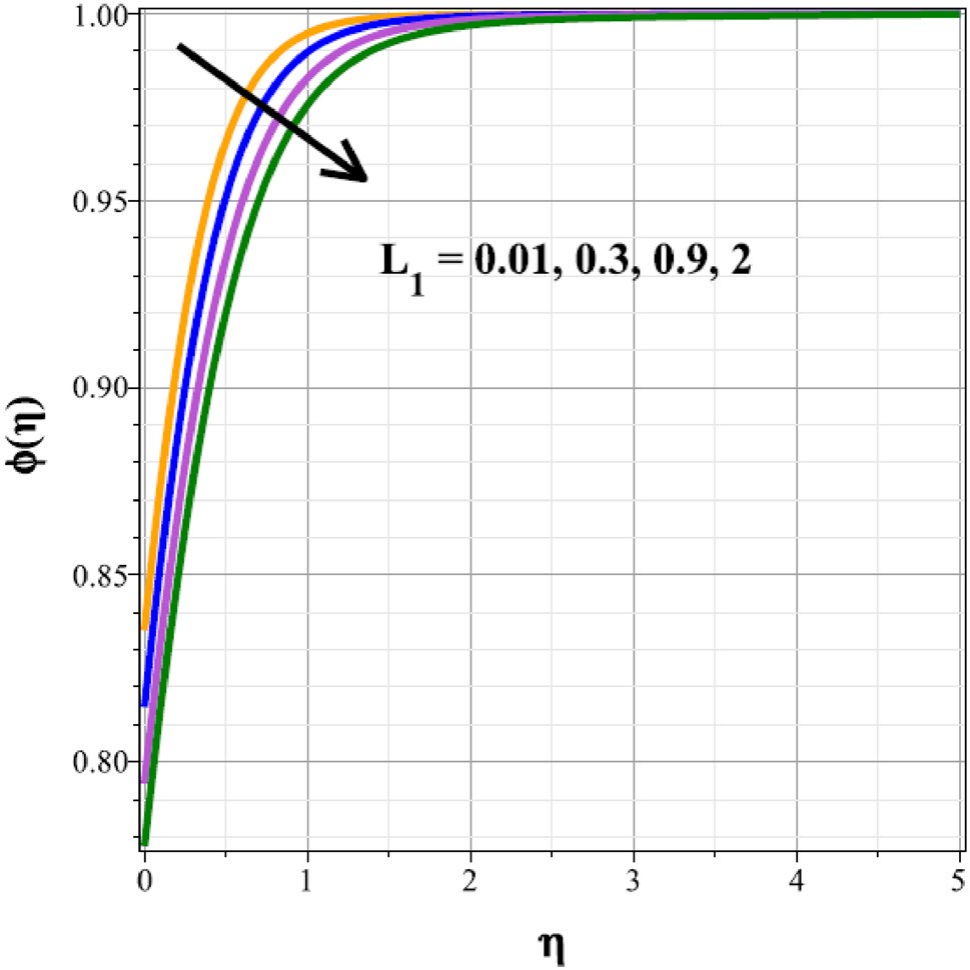
Curves of $\phi (\eta )$ for ${L}_{1}$.

To identify the distinctive differences in the solutal regime for the components of the unsteady factor $({\delta }^{*})$, homogenic reaction strength $({k}_{1})$, heterogenic reaction strength $({k}_{2})$ and velocity slip factor $({L}_{1})$, Figs. 16, 17, 18 and 19 are successively detailed. The mass movement in the flow is impeded by the flow's enhanced unsteadiness. As a result, mass distribution slows down as ${\delta }^{*}$ increases in magnitude, as seen in Fig. 16. Figure 17 shows the concentration panel's curves for the upshot values of ${k}_{1}$. The concentration profile is discouraged by a rise in ${k}_{1}$. The link between the concentration panel and ${k}_{2}$ is further explained in Fig. 18. Due to an increase in mass diffusion, a rising influence of ${k}_{2}$ deescalates the mass transfer profile. The influence of the velocity slip parameter on the mass transfer regime is shown as a result in Fig. 19. Here, when ${L}_{1}$ increases, the mass transfer panel decreases.

**Figure 20:**
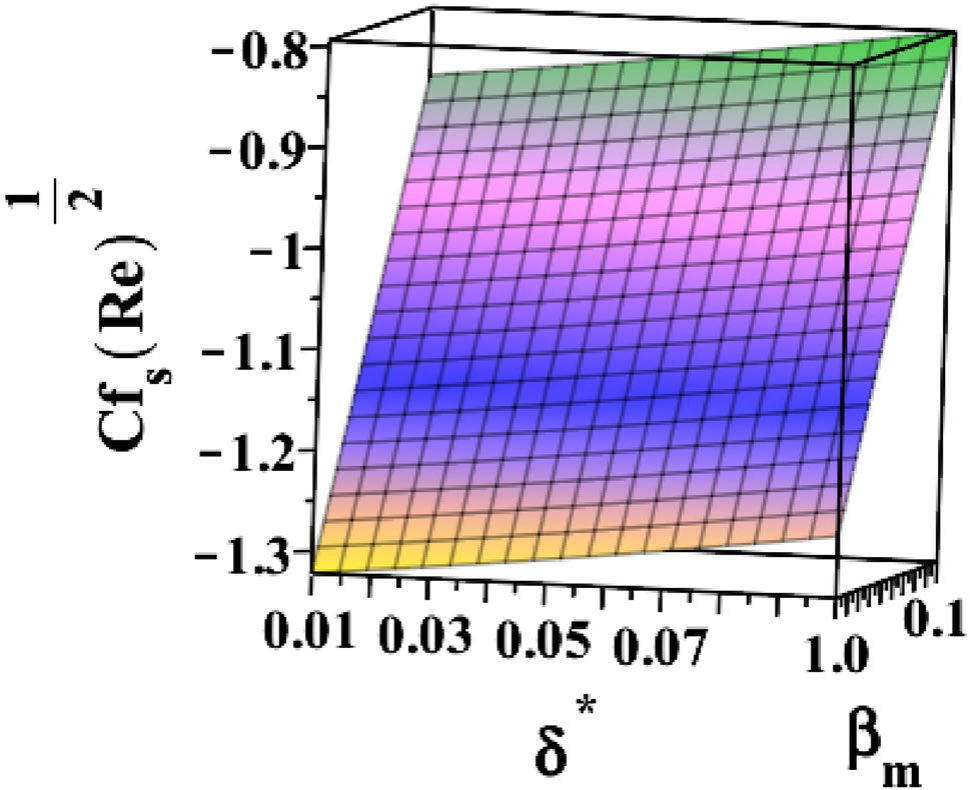
3D depiction of ${\varvec{C}}{{\varvec{f}}}_{{\varvec{s}}}{\left({\varvec{R}}{\varvec{e}}\right)}^\frac{1}{2}$ for ${\delta }^{*}$ against ${\beta }_{m}$.

**Figure 21:**
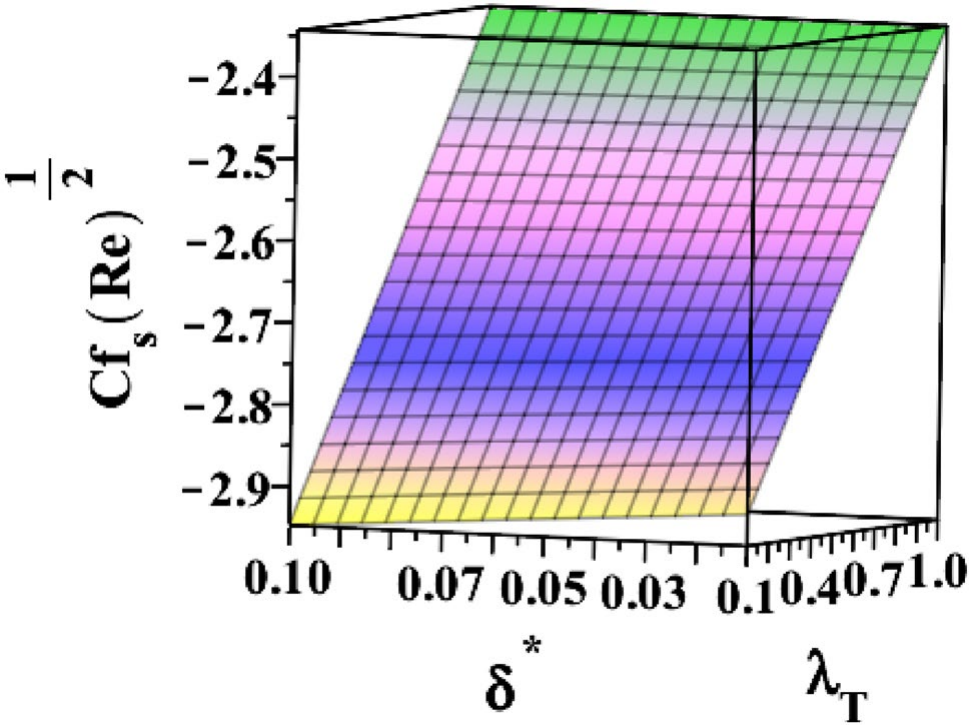
3D depiction of ${\varvec{C}}{{\varvec{f}}}_{{\varvec{s}}}{\left({\varvec{R}}{\varvec{e}}\right)}^\frac{1}{2}$ for ${\delta }^{*}$ against ${\lambda }_{T}$.

**Figure 22:**
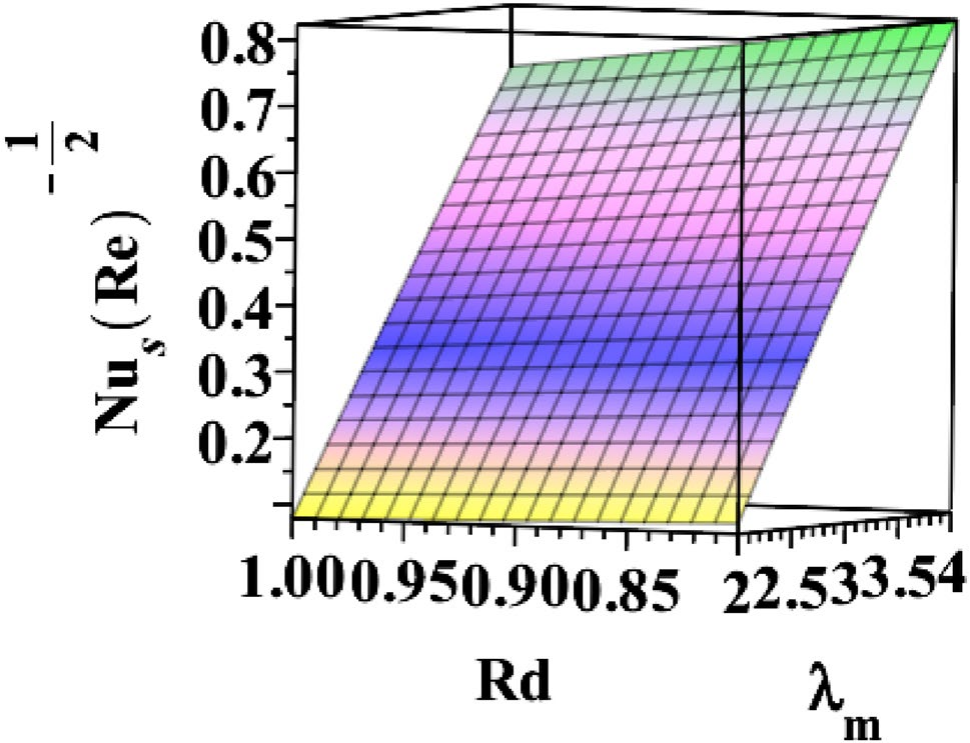
3D depiction of ${\varvec{N}}{{\varvec{u}}}_{{\varvec{s}}}{\left({\varvec{R}}{\varvec{e}}\right)}^{-\frac{1}{2}}$ for $Rd$ against ${\lambda }_{m}$.

**Figure 23:**
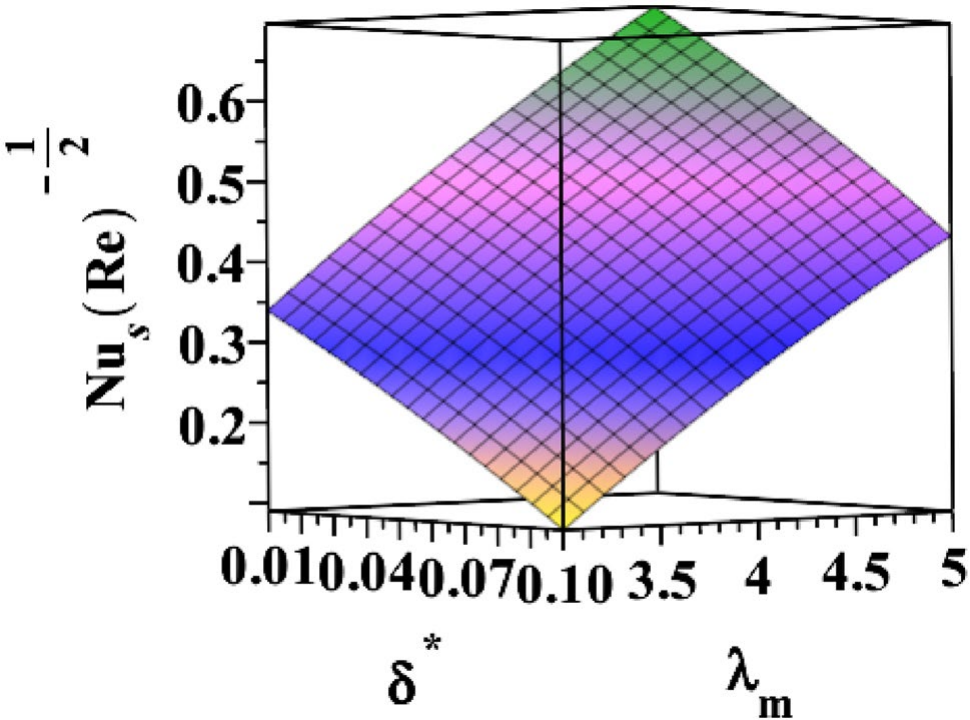
3D depiction of ${\varvec{N}}{{\varvec{u}}}_{{\varvec{s}}}{\left({\varvec{R}}{\varvec{e}}\right)}^{-\frac{1}{2}}$ for ${\delta }^{*}$ against ${\lambda }_{m}$.

The 3D skin friction coefficient is represented against the unsteadiness parameter ${(\delta }^{*})$ in Fig. 20 for changing ferrohydrodynamic interaction parameter ${(\beta }_{m})$. Skin friction co-efficient increased due to the unsteadiness parameter's amplification. Every time the ferrohydrodynamic interaction parameter increases, the surface drag decreases. Figure 21 explains how the unsteadiness parameter behaves in relation to the thermal buoyancy parameter ${(\lambda }_{T})$. Surface drag is shown to decrease as the unsteadiness parameter $({\delta }^{*})$ approaches its maximum value. The skin friction coefficient increases with the thermal buoyancy parameter. Figure 22 illustrates how the radiation parameter $(Rd)$ responds to the heat dissipation factor $({\lambda }_{m})$ on Nusselt number $\left(N{u}_{S}R{e}^{-\frac{1}{2}}\right)$. The heat transfer rate marginally decreases when the sheet is exposed to intense radiation. The rate is also slightly increased by magnifying $({\lambda }_{m})$. The higher the heat dissipation factor number, the greater the rate of heat transmission. Figure 23 illustrates the characteristics of the unsteadiness parameter ${(\delta }^{*})$ for different heat dissipation factors $({\lambda }_{m})$. Increases in the unsteadiness parameter ${(\delta }^{*})$ result in the lowest heat transfer rate.

**Figure 24:**
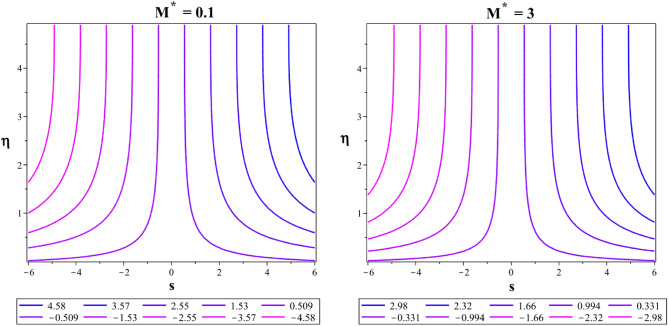
Flow streamlines at ${M}^{*}=0.1$ and ${M}^{*}=3$.

**Figure 25:**
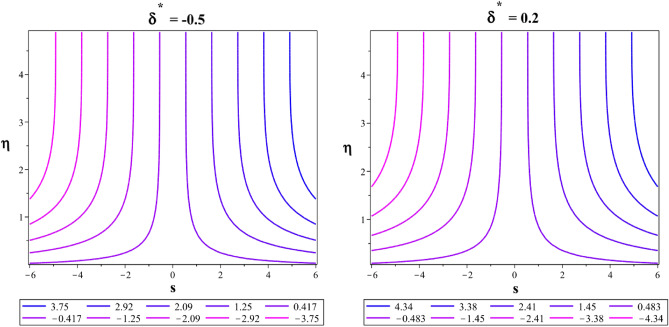
Flow streamlines at ${\delta }^{*}=-0.5$ and ${\delta }^{*}=0.2$.

**Figure 26:**
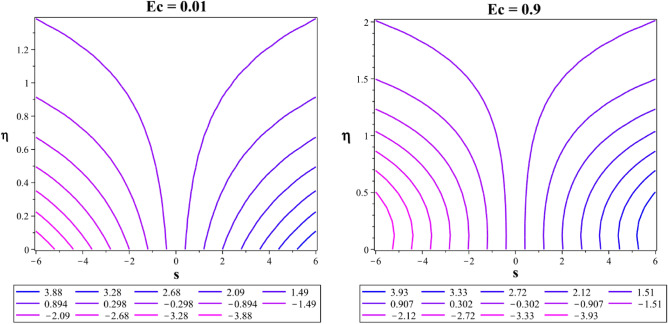
Flow isotherms at ${\varvec{E}}{\varvec{c}}=0.01$ and ${\varvec{E}}{\varvec{c}}=0.9$.

In a 2D contour plot (streamlines) Fig. 24, the trajectory followed by the Casson-Williamson fluid particles is described for magnetic parameter at ${M}^{*}=0.1$ and ${M}^{*}=3$. In Fig. 25, the streamlines for the unsteadiness parameters ${\delta }^{*}=-0.5$ and ${\delta }^{*}=0.2$ are also explained. Figure 26 shows a plot with contours (isotherms) showing similar temperatures at places over a stretched area for $Ec=0.01$ and $Ec=0.9$.

## Concluding reviews

After all considerations and parametric study on each solution graph, the following list summarizes key findings from the current study:

- Increase in the ferrohydrodynamic interaction factor $\left({\beta }_{m}\right)$ and velocity slip factor $\left({L}_{1}\right)$ obstruct the velocity profile $({f}{\prime}\left(\eta \right))$, whereas the thermal buoyancy factor $\left({\lambda }_{T}\right)$ promotes it.
- The unsteady factor's $(\epsilon )$ velocity profile (${f}^{\prime}(\eta )$) has a dual character, decreasing close to the sheet and rising distant from the sheet.
- When the thermal buoyancy factor $\left({\lambda }_{T}\right)$ and melting heat parameter increase ($Me$), the thermal distribution ($\theta (\eta )$) is drained.
- In terms of thermal distribution ($\theta (\eta )$), the Curie temperature $(\epsilon )$ exhibits a dual nature, increasing close to the sheet and decreasing far from the sheet.
- With growing homo-heterogenic strengths (${k}_{1}\,\, and\,\, {k}_{2})$ and an unsteadiness parameter $({\delta }^{*})$, the mass distribution $(\phi (\eta ))$ exhibits negative behaviour.

## Data Availability

The datasets used and/or analysed during the current study available from the corresponding author on reasonable request.
