# Author Correction: Magnetic dipole effects on unsteady flow of Casson-Williamson nanofluid propelled by stretching slippery curved melting sheet with buoyancy force

**DOI:** 10.1038/s41598-023-40642-3

**Published:** 2023-08-21

**Authors:** Pradeep Kumar, Basavarajappa Nagaraja, Felicita Almeida, Abbani Ramakrishnappa AjayKumar, Qasem Al‑Mdallal, Fahd Jarad

**Affiliations:** 1 Department of Mathematics, School of Engineering, Presidency University, Rajanakunte, Yelahanka, Bengaluru, Karnataka 560064 India; 2 Department of Mathematics, KNS Institute of Technology, Thirumenahalli, Yelahanka, Bangalore, 560064 India; 3 Department of Mathematical Sciences, UAE University, P.O. Box 17551, Al‑Ain, United Arab Emirates; 4 Department of Mathematics, Çankaya University, 06790 Ankara, Turkey; 5 Department of Medical Research, China Medical University, Taichung, 40402 Taiwan

Correction to: *Scientific Reports* https://doi.org/10.1038/s41598-023-39354-5, published online 07 August 2023

The original version of this Article omitted an affiliation for Fahd Jarad. Their correct affiliations are listed below.

Department of Mathematics, Çankaya University, 06790, Ankara, Turkey.

Department of Medical Research, China Medical University, Taichung, 40402, Taiwan.

The original Article has been corrected.

